# Selection of Suitable Reference Genes for RT-qPCR Normalization under Abiotic Stresses and Hormone Stimulation in Persimmon (*Diospyros kaki* Thunb)

**DOI:** 10.1371/journal.pone.0160885

**Published:** 2016-08-11

**Authors:** Peihong Wang, Aisheng Xiong, Zhihong Gao, Xinyi Yu, Man Li, Yingjun Hou, Chao Sun, Shenchun Qu

**Affiliations:** 1 College of Horticulture, Nanjing Agricultural University, Nanjing, 210095, China; 2 State Key Laboratory of Crop Genetics and Germplasm Enhancement, College of Horticulture, Nanjing Agricultural University, Nanjing, 210095, China; Louisiana State University College of Agriculture, UNITED STATES

## Abstract

The success of quantitative real-time reverse transcription polymerase chain reaction (RT-qPCR) to quantify gene expression depends on the stability of the reference genes used for data normalization. To date, systematic screening for reference genes in persimmon (*Diospyros kaki* Thunb) has never been reported. In this study, 13 candidate reference genes were cloned from 'Nantongxiaofangshi' using information available in the transcriptome database. Their expression stability was assessed by geNorm and NormFinder algorithms under abiotic stress and hormone stimulation. Our results showed that the most suitable reference genes across all samples were *UBC* and *GAPDH*, and not the commonly used persimmon reference gene *ACT*. In addition, *UBC* combined with *RPII* or *TUA* were found to be appropriate for the "abiotic stress" group and *α-TUB* combined with *PP2A* were found to be appropriate for the "hormone stimuli" group. For further validation, the transcript level of the *DkDREB2C* homologue under heat stress was studied with the selected genes (*CYP*, *GAPDH*, *TUA*, *UBC*, *α-TUB*, and *EF1-α*). The results suggested that it is necessary to choose appropriate reference genes according to the test materials or experimental conditions. Our study will be useful for future studies on gene expression in persimmon.

## Introduction

Persimmon (*Diospyros kaki* Thunb.), which is prevalent worldwide, originated in Eastern Asia and was mainly cultivated in China, Korea, and Japan [1]. Due to lack of genetic or genomic information, previous studies on persimmon mainly focused on its diversity and phylogeny [2]. Currently, with the development of genomic technologies, molecular biology studies on persimmon have gained attention and provide further understanding of complex biological mechanisms in persimmon. For example, sequence-specific amplification polymorphism (SSAP) was applied to reveal the genes associated with deastringency in persimmon [3]; genome-wide transcriptome analysis was performed to identify the primary genes involved in proanthocyanidin (PA) biosynthesis in persimmon [2]. For these molecular biological studies, gene expression analysis is an effective and widely used approach commonly performed by relying on methods such as Northern blotting, microarray analysis, and quantitative real-time reverse transcription polymerase chain reaction (RT-qPCR).

Rapidity, sensitivity, specificity, and quantification are relevant features of RT-qPCR [4,5] that make it the preferred method for gene expression analyses. However, when performing relative quantitative experiments, RNA quantity, quality, and processing may influence the accuracy of the results [6]. To ensure the accuracy of RT-qPCR among different samples, the choice of one or more verified reference genes is particularly critical [7–9]. The most commonly used RT-qPCR reference genes are housekeeping genes, which act as a basic component of the organelle skeleton or participate in the basic biochemical metabolism of the organism. Thus, it is commonly believed that these genes are not regulated or influenced by environmental and growth factors. These genes include: *ACT* (actin), *GAPDH* (glyceraldehyde-3-phosphate dehydrogenase), *β-TUB* (beta tubulin), *α-TUB* (alpha tubulin), *UBQ* (polyubiquitin), *18S rRNA* (18S ribosomal RNA), etc. [10,11]. However, several studies have indicated that their expression levels do not remain relatively stable under different conditions in different species [12–16]. In other words, the so-called constant expression of these genes is only constant under certain conditions or species. It is thus clear that screening appropriate reference genes according to the test materials or experimental conditions is important. In recent years, related studies have increased in non-model species, such as *Oenanthe javanica* [17], *Caragana intermedia* [8], carrot [18], watermelon [19], pepper [20], oil palm [21], celery [22], and *Heterosigma akashiwo* [23]. To date, no systematic screening for reference genes has been reported in persimmon.

In this study, 13 candidate reference genes (*ACT*, *α-TUB*, *β-TUB*, *UBC*, *CYP*, *RPL13*, *PP2A*, *GAPDH*, *EF1-α*, *F-box*, *RPII*, *TUA*, and *SAND*) were selected because of their stable expression as evidenced in previous studies [17,24–26]. Their sequences were obtained from the transcriptome sequencing database of persimmon. Their stabilities in persimmon were analyzed by two algorithms (geNorm and NormFinder) under abiotic stresses (heat, cold and salt) and hormone stimulation, including gibberellin, salicylic acid and abscisic acid. Furthermore, the reliability of the identified reference genes was verified by assessing the expression level of the *DkDREB2C* gene under heat treatment. This work aims to select suitable reference genes for future gene expression studies under abiotic stresses and hormone stimulation in persimmon.

## Materials and Methods

### Plant Materials and Treatments

`Nantongxiaofangshi' (*D*. *kaki* Thunb.) is a good persimmon cultivar suitable for dwarfing and closely spaced planting due to weak apical dominance and no obvious trunk [27]. In this study, virus-free clonal persimmon (*D*. *kaki* Thunb. cv. Nantongxiaofangshi) microplants were obtained from the College of Horticulture, Nanjing Agricultural University. Seedlings were grown on MS propagation medium with 1 mg/L ZT and 0.1 mg/l IAA (pH = 6.8–7.0) at a constant temperature of 25°C and a 16:8 h light:dark cycle with an illumination of 4100 lx. There were three biological experimental replicates per treatment. The control group was grown in normal medium and conditions as stated above.

For the hormone treatments, 100 μM gibberellins (GA treatment) [28], 100 μM salicylic acid (SA treatment) [29], or 100 μM abscisic acid (ABA treatment) [30] was added to the media. Then, three-week-old seedlings on the normal medium were transplanted into the medium with hormone for a week. Salt treatment (150 mM NaCl) was applied in the same way [31,32]. Temperature treatments included placing three-week-old tissue culture seedlings in light incubators at 4°C (cold treatment) or 40°C (heat treatment) for 6 h. Leaves were collected from both the treated samples and the control group. The samples were immediately immersed in liquid nitrogen and stored at −80°C for further use.

### RNA Extraction and cDNA Synthesis

Frozen samples were ground to a fine powder in liquid nitrogen and total RNA was extracted using Plant Total RNA Isolation Kit Plus (Foregene, Chengdu, China). RNA integrity was checked by 1% agarose gel electrophoresis and RNA quality was measured by Nanodrop ND 1000 spectrophotometer (Nanodrop Technologies Inc, Delaware, USA). Samples with 28S/18S ribosomal RNA between 1.5 and 2.0 and an absorbance ratio OD_260_/_280_ between 1.9 and 2.2 were used for subsequent experiments. Approximately 1,000 ng total RNA was used for cDNA synthesis using the PrimeScript RT reagent Kit with gDNA Eraser (TaKaRa, Dalian, China). The cDNA was diluted with nuclease-free water before RT-qPCR.

### Selection of Candidate Reference Genes

In our study, the stability of 13 candidate reference genes was evaluated. Among these genes, *ACT* is commonly used in persimmon [2,3,33–35] and others have been validated as good reference genes in other crops [8,17–21,26,36,37], including α*-TUB*, *β-TUB*, *UBC*, *CYP*, *RPL13*, *PP2A*, *GAPDH*, *EF1-α*, *F-box*, *RPII*, *TUA*, and *SAND*. Based on the transcriptome sequencing data of ‘Nantongxiaofangshi’ leaves (unpublished data), potential homologues of the 13 reference genes were cloned and identified. Meanwhile, the corresponding target amplicon of each gene was sequenced (Biogene, Nanjing, China). Details were presented in Table 1, S1 Table and S3–S15 Figs.

**Table 1:** Primer information of reference genes in persimmon (qPCR).

Gene symbol	Gene name	Arabidopsis homolog locus	Primer sequence (5'–3')	Amplicon length (bp)	Tm (°C)	E (%)
*ACT*	Actin 2 gene	AT5G09810	CTGGATTCTGGTGATGGT/GCAGTTGTTGTGAAGGAG	155	82.2	108.5
*α-TUB*	Tubulin alpha-4	AT5G19780	TCTCCACCTCTGTTGTTG/CGTAGATCGCCTCGTTAT	102	78.8	105.5
*β-TUB*	Tubulin beta-1	AT1G75780	TCCTGGTCAACTCAACTC/CTGTAAGGGCACGGTATT	131	80.4	95.9
*UBC*	Ubiquitin-conjugating enzyme	AT1G64230	CCTCACGACAACAACATC/ATATACTCCCATCCGCATAG	181	79.7	107.7
*CYP*	Cyclophilin	AT3G63400	CGGATCGCAATTCTTCATC/GCAATCAGCAATGGTCAC	160	84.8	104.9
*RPL13*	60S ribosomal protein L13-1	AT5G23900	GACTAATGTCCAGAGGTTGA/GCTTCTCACGCACAATAG	152	80.7	105.2
*PP2A*	Protein phosphatase 2A	AT4G15415	CGGTGCTTATCATCAACAG/GCTCAAGAACATCAACTCC	139	79.2	100.9
*GAPDH*	Glyceraldehyde-3- phosphate dehydrogenase gene	AT1G42970	TATTCCTAGCAGCACTGG/ATATGTAGCCGCCTTCTC	148	80.8	100.4
*EF 1-α*	Elongation factor -1αgene	AT1G07940	CTGACTGTGCTGTTCTTATC/GTGGCATCCATCTTGTTG	148	80.3	98.3
*F-box*	F-box/kelch-repeat protein	AT5G15710	GTATTGCCTTGCTCTTGTC/CCTTGCCTTCACTATCCA	164	80.3	104.6
*RPII*	DNA-directed RNA polymerase II	AT2G15430	TCCTGAGGCATACACATAC/CATTGAGCACCAACTGAG	152	78	99.8
*TUA*	Tubulin alpha-3/alpha-5 chain	AT5G19780	AGCCCTCAAGTATGATGG/GCCTACAGCAGCATTAAC	110	78.9	103.9
*SAND*	SAND family protein gene	AT2G28390	GCAGGATTCGTATTGAGATG/CTGAGCGAGAAGATGGAT	120	78.1	102.3

### PCR Primer Design and Amplification Efficiency Testing

Using the BioEdit Sequence Alignment v 7.0.9 software, the potential homolog sequences of these genes were aligned and edited. The primers for RT-qPCR were designed using the Beacon Designer 7.0 software and the primers for cloning were designed online (NCBI/Primer-Blast). The PCR products of the expected size were sequenced to confirm the specificity of these genes. For all primer pairs used for RT-qPCR, the optimal annealing temperature and amplification specificity were separately tested by gradient PCR and 1.5% gel electrophoresis. The efficiency (E) for these primer sets, which amplified a single, specific product, was estimated by standard curve analysis of five-fold diluted series (1, 5, 5^2^, 5^3^, 5^4^, and 5^5^ × dilution) with the following equation: E = [10^(-1/slope)^-1]×100%, where the slope is the standard curve slope. The information about the specificity-validated primers was summarized in Table 1.

### Quantitative Real-Time PCR Assay

**Fig 1:**
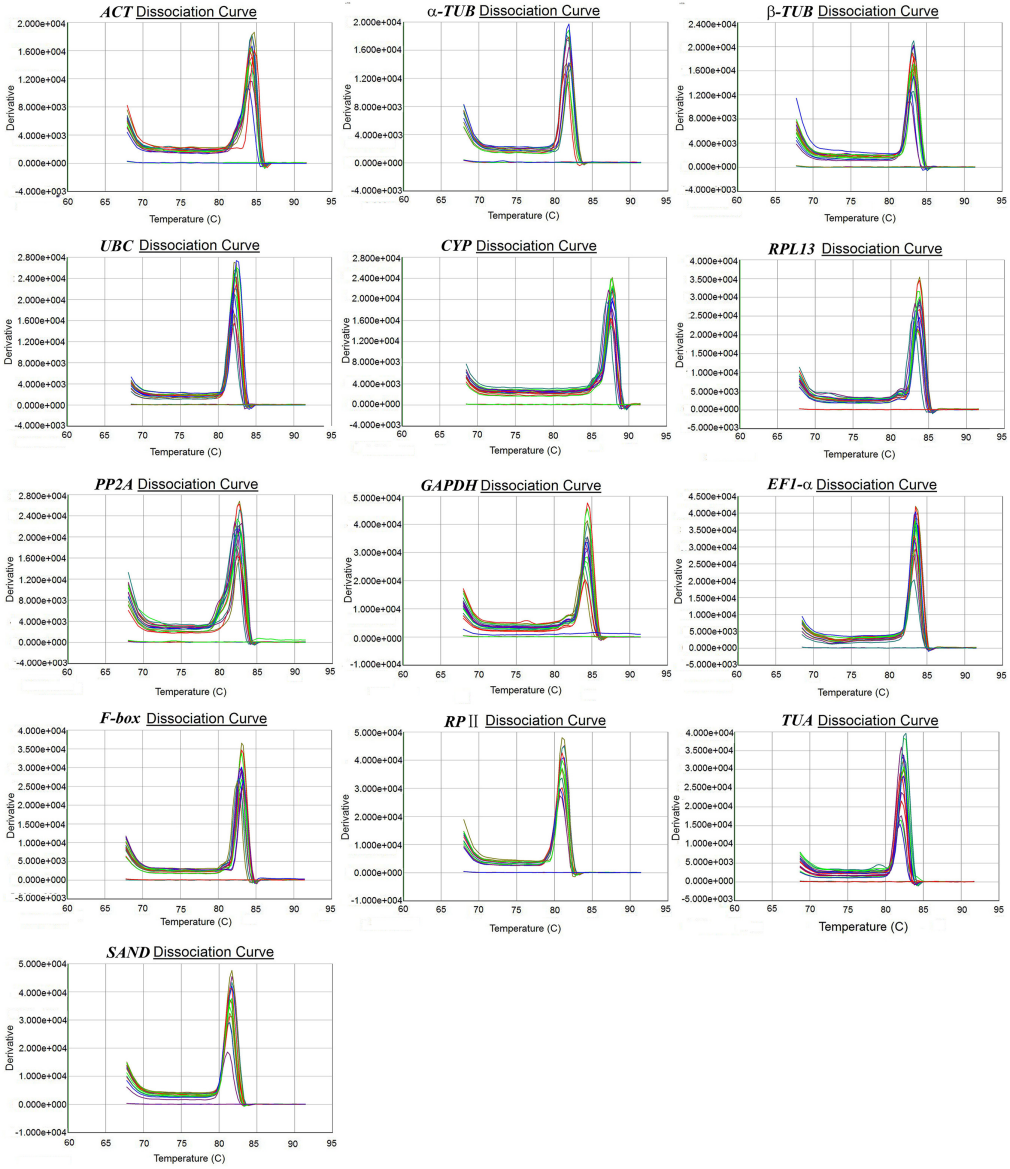
Melting curves of the 13 candidate reference genes. In order: *ACT*, α*-TUB*, *β-TUB*, *UBC*, *CYP*, *RPL13*, *PP2A*, *GAPDH*, *EF1-α*, *F-box*, *RPII*, *TUA*, and *SAND*.

RT-qPCR reactions were performed by using an ABI 7300 Real-Time PCR System (Applied Biosystems, USA) with SYBR Premix *Ex Taq*^™^ (TaKaRa, Dalian, China). The 20 μL reaction system consists of 10 μL SYBR Green I Mix, 1 μL diluted cDNA template (140 ng/uL), 8.6 μL ddH_2_O, and 0.2 μL each primer. The following cycling conditions were used: an initial denaturation step of 95°C (30 s), followed by 40 cycles of 95°C (5 s) and 60°C (30 s). Dissociation curves were generated from 60°C to 95°C to verify primer specificity with the presence of a single peak (Fig 1). Meanwhile, there were three technical and biological replicates, as well as a no-template control in all assays. All RT-qPCR reactions were carried out in accordance with the Minimum Information for Publication of Quantitative Real-Time PCR Experiments (MIQE) guidelines [38].

### Data Analysis

Two different types of statistical algorithms, geNorm [39] and NormFinder [40], were employed to rank the expression stability of 13 candidate reference genes under the different experimental conditions. The quantification cycle (Cq) values from the RT-qPCR were listed in S2 Table. Before the raw Cq values were input into the software mentioned above, all Cq values were converted to relative quantities by using the formula: 2^- ΔCq^, in which ΔCq = the corresponding Cq value—minimum Cq.

In geNorm, the reference gene expression stability measurement (M) value is automatically calculated. The expression ratio of two ideal internal control genes is identical in a given sample set, which is considered as the principle of the program. Regardless of the co-regulation of the control genes, a combination of two housekeeping genes with the most stable expression level in the tested samples was verified by stepwise exclusion of the least stable gene with the highest M value [39]. Normfinder enables avoidance of misinterpretations caused by artificial selection of co-regulated genes [18]. Relying on intra- and inter-group variations, this program ranks all candidate reference genes and combines both results to yield a stability value for each candidate reference gene [40].

## Results

### Primer Specificity and Amplification Efficiencies

The specificity of each primer pair was verified by a single band, which had the expected size in agarose gel electrophoresis (S1 Fig). The absence of both signals in the no-template controls and primer dimers further confirmed the specificity of the primers (Fig 1). The amplification efficiency (E) of the 13 candidate reference genes ranged from 95.9%-108.5% (Table 1), which was calculated from the standard curves with good linear relationships (R^2^ varying from 0.990–0.996) (S2 Fig). Both E and R^2^ were within the commonly reported range for RT-qPCR.

### Expression Profiling of Candidate Reference Genes

**Fig 2:**
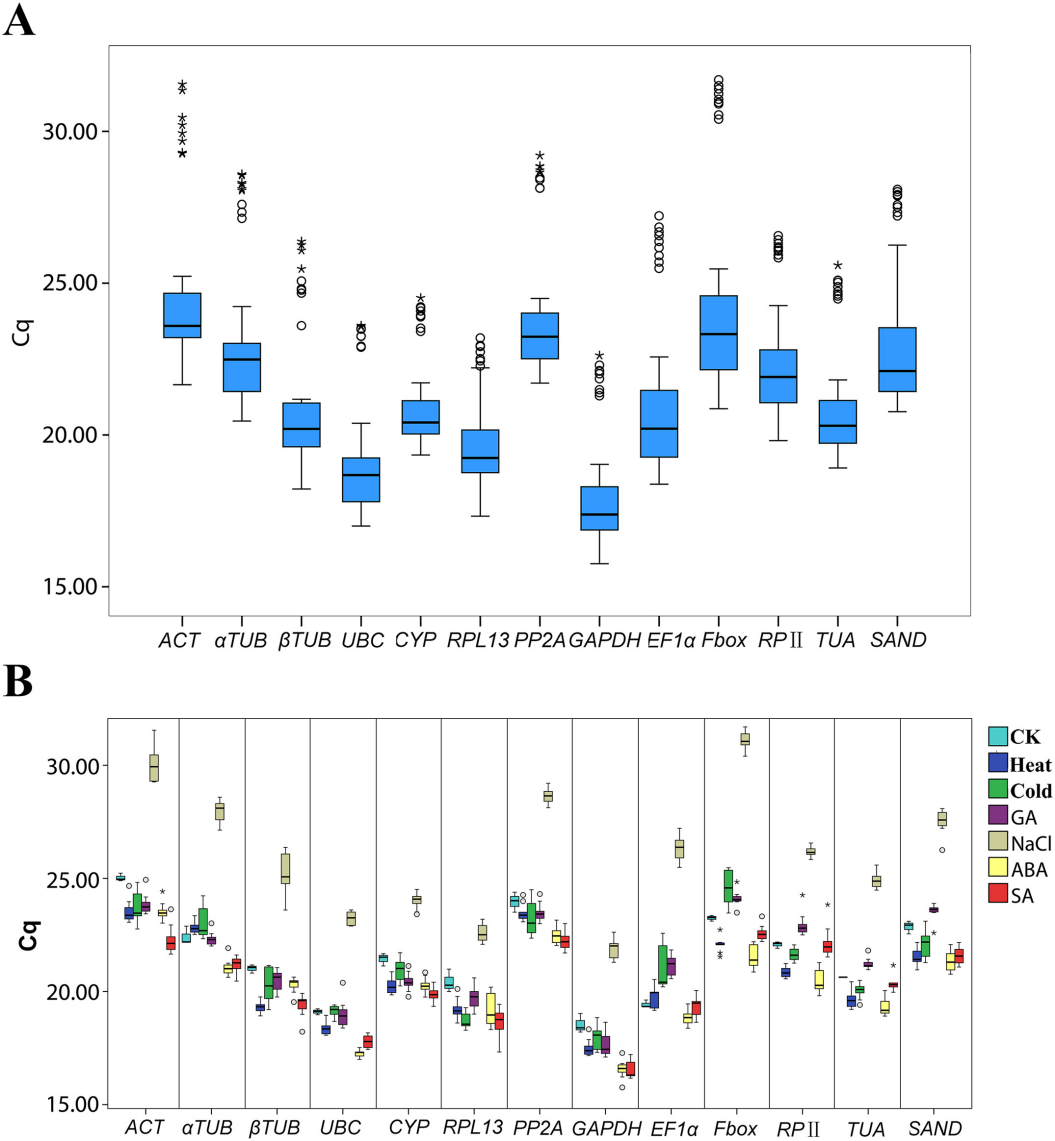
Expression levels of the candidate reference genes in persimmon. A: Cq values for the reference genes across all samples. B: Cq values of each reference gene in the single treatment and control group (CK). Boxes depict Cq values, including the median values (lines across the boxes), Q1 (one-quarter, lower outline), Q3 (three-quarters, upper outline), and whiskers. The whiskers are set at Q3+1.5IQR (interquartile range) and Q1-1.5IQR. Mild outliers (°) represent the values outside this range but within Q3+3IQR and Q1-3IQR. Extreme outliers (*) represent the values outside Q3+3IQR or Q1-3IQR.

The expression level of the 13 candidate genes was determined as quantification cycle (Cq) values (S2 Table). The variations of their transcript levels were clearly shown by the box-plot (Fig 2). As shown in Fig 2A, *GAPDH* exhibited the highest expression level with lower Cq values (ranging from 15.76 to 22.62, S2 Table) than the other genes, whereas *ACT* and *F-box* had higher Cq values (ranging from 21.66 to 31.55 and from 20.86 to 31.70, respectively, S2 Table). Furthermore, there were many outliers (mild and extreme) for each gene across all samples, which affected the judgment of their expression stability. To directly observe the distribution of these outliers, a distribution diagram of each gene in a single treatment and the control group was drawn (Fig 2B). The Cq values of all genes under salt treatment were obviously higher than in the other five treatments. That is, all of the tested reference genes showed a low expression level in salt treatment. *CYP* was the least variable gene among the remaining samples. However, *EF1-α* and *F-box* were the most variable genes.

### Expression Stability Analyses

After a simple comparison of the raw Cq values, geNorm and NormFinder were used to further evaluate the stability of the candidate reference genes. In addition, we sorted six different treatment sets into three groups: "abiotic stress" (heat, cold and salt), "hormone stimuli" (SA, GA and ABA) and "total" (samples in all treatments). For both the single stress treatments and groups, 9 evaluation patterns were generated.

#### a) geNorm analysis

**Fig 3:**
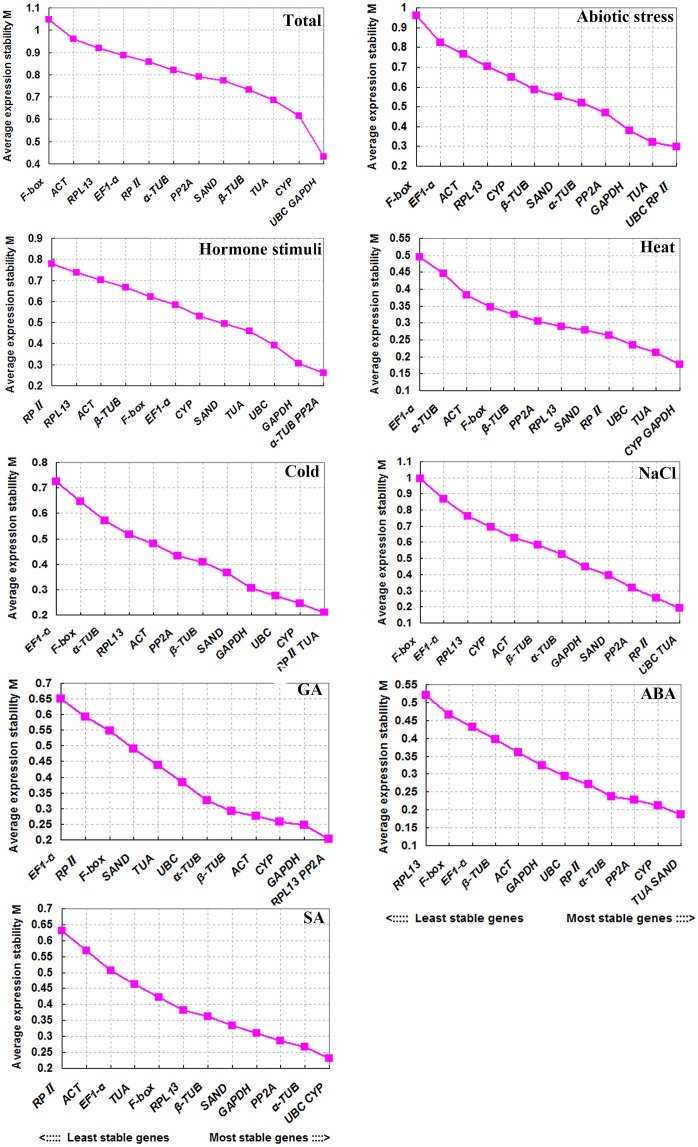
Ranking of the expression stability of the 13 reference genes in geNorm. The tested genes were ranked according to their expression stability by stepwise exclusion of the gene with the highest M value. The gene with the highest M value is on the left and is the least stable. Conversely, the gene on the right is the most stable.

geNorm generated the ranks of the selected reference genes based on the expression stability value M, which were shown in Fig 3. By graphing the results, it was not difficult to determine that all of the genes performed well in both individual stress conditions and multiple stresses, with M values less than the default limit (1.5).

In heat treatment, *CYP* and *GAPDH* were the most stable genes and *CYP* was also one of the top-ranked genes in SA treatment. The other best gene in SA treatment was *UBC*, which ranked as one of the top two under NaCl treatment, abiotic stress, and total. In cold treatment, *RPII* and *TUA* ranked in the top two. *TUA* and *SAND* were the highest-ranked genes in ABA treatment. *α-TUB* and *PP2A* were the best-performing genes in the “hormone stimuli” group. Among all treatments and groups, *EF1-α*, *F-box*, *RPL13*, and *ACT* showed relative instability, especially *EF1-α* and *F-box*.

**Fig 4:**
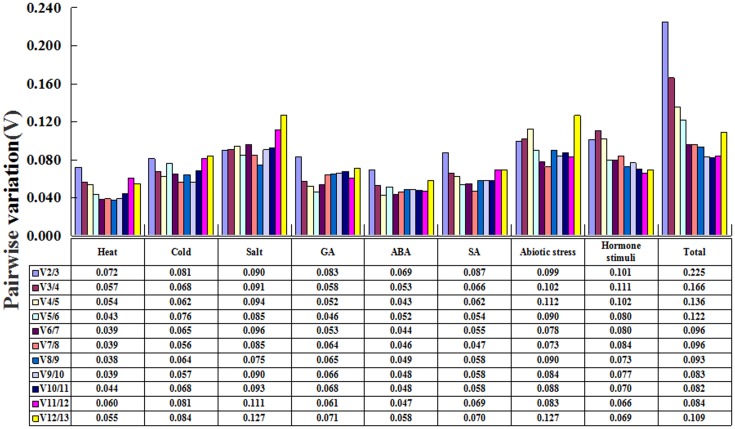
Determination of the optimal number of reference genes required for effective normalization. By using geNorm, the optimal number of reference genes in each sample was determined by an analysis of pairwise variation (V_n/n+1_) between the normalization factors (NF_n_ and NF_n +1_).

In addition, we determined the optimal number of reference genes using geNorm with the calculation of pairwise variations (V_n/n+1_) between the sequential normalization factors (NF_n_ and NF_n+1_, n ≥ 2) [39]. As Fig 4 shows, except for the “total” group, the inclusion of the third gene was not required for the remaining groups and treatments with a low V_2/3_ value, which was below the cut-off value (0.15) [39]. In other words, two reference genes would be sufficient to normalize gene expression under these conditions. However, four genes were needed for the ''total'' group (V_4/5_ = 0.136).

#### b) NormFinder analysis

NormFinder was used to perform an independent assessment of these reference genes. The rankings were listed in Tables 2 and 3. According to NormFinder, except for ABA stress, *UBC* performed well with a ranking in the top three under the remaining individual stress conditions and three groups. For individual experimental treatments, *UBC* was the most stable gene for the heat, cold and SA treatments; *α-TUB* was the best reference gene for the ABA and GA stresses; and *PP2A* ranked first in the NaCl treatment. Considering all the conditions together, *UBC* was the most stable genes according to both software algorithms. *EF1-α*, *F-box*, *ACT*, and *RPL13* were low-ranking genes in most single stresses and groups, which was consistent with the result from geNorm analysis. Thus, these genes might not be suitable for use as reference genes.

**Table 2:** Gene expression stability under individual treatment ranked by NormFinder software.

Rank	Heat		Cold		NaCl		ABA		GA		SA	
	Gene	Stability	Gene	Stability	Gene	Stability	Gene	Stability	Gene	Stability	Gene	Stability
1	*UBC*	0.090	*UBC*	0.179	*PP2A*	0.118	*α-TUB*	0.105	*α-TUB*	0.103	*UBC*	0.085
2	*TUA*	0.126	*CYP*	0.189	*SAND*	0.168	*ACT*	0.116	*UBC*	0.118	*α-TUB*	0.094
3	*GAPDH*	0.131	*GAPDH*	0.191	*UBC*	0.183	*TUA*	0.120	*PP2A*	0.261	*SAND*	0.125
4	*CYP*	0.169	*TUA*	0.209	*TUA*	0.203	*PP2A*	0.132	*β-TUB*	0.263	*CYP*	0.195
5	*F-box*	0.204	*β-TUB*	0.228	*β-TUB*	0.282	*CYP*	0.141	*TUA*	0.278	*β-TUB*	0.220
6	*RPL13*	0.212	*RPII*	0.230	*ACT*	0.304	*SAND*	0.144	*RPL13*	0.350	*PP2A*	0.257
7	*PP2A*	0.230	*PP2A*	0.283	*RPII*	0.325	*RPII*	0.201	*SAND*	0.369	*F-box*	0.291
8	*RPII*	0.233	*SAND*	0.294	*α-TUB*	0.443	*UBC*	0.201	*CYP*	0.398	*RPL13*	0.330
9	*SAND*	0.243	*ACT*	0.419	*GAPDH*	0.509	*F-box*	0.204	*GAPDH*	0.410	*GAPDH*	0.346
10	*ACT*	0.283	*α-TUB*	0.435	*CYP*	0.772	*RPL13*	0.224	*F-box*	0.419	*TUA*	0.417
11	*β-TUB*	0.321	*RPL13*	0.611	*EF1-α*	0.870	*GAPDH*	0.275	*ACT*	0.445	*EF1-α*	0.450
12	*EF1-α*	0.489	*F-box*	0.667	*RPL13*	0.967	*β-TUB*	0.279	*RPII*	0.477	*ACT*	0.587
13	*α-TUB*	0.510	*EF1-α*	0.722	*F-box*	1.234	*EF1-α*	0.309	*EF1-α*	0.662	*RPII*	0.620

**Table 3:** Gene expression stability under multiple stresses ranked by NormFinder software.

Rank	Total		Abiotic stress		Hormone stimuli	
	Gene	Stability	Gene	Stability	Gene	Stability
1	*UBC*	0.215	*TUA*	0.181	*α-TUB*	0.089
2	*PP2A*	0.273	*UBC*	0.202	*UBC*	0.185
3	*SAND*	0.288	*RPII*	0.242	*PP2A*	0.210
4	*GAPDH*	0.316	*PP2A*	0.243	*GAPDH*	0.271
5	*TUA*	0.327	*β-TUB*	0.268	*SAND*	0.307
6	*β-TUB*	0.341	*SAND*	0.286	*CYP*	0.325
7	*CYP*	0.427	*GAPDH*	0.343	*TUA*	0.330
8	*α-TUB*	0.435	*α-TUB*	0.361	*β-TUB*	0.385
9	*ACT*	0.484	*ACT*	0.481	*F-box*	0.418
10	*EF1-α*	0.496	*CYP*	0.559	*RPL13*	0.424
11	*RPII*	0.504	*EF1-α*	0.616	*EF1-α*	0.448
12	*RPL13*	0.520	*RPL13*	0.724	*ACT*	0.508
13	*F-box*	0.667	*F-box*	0.999	*RP II*	0.560

### Reference Gene Validation

**Fig 5:**
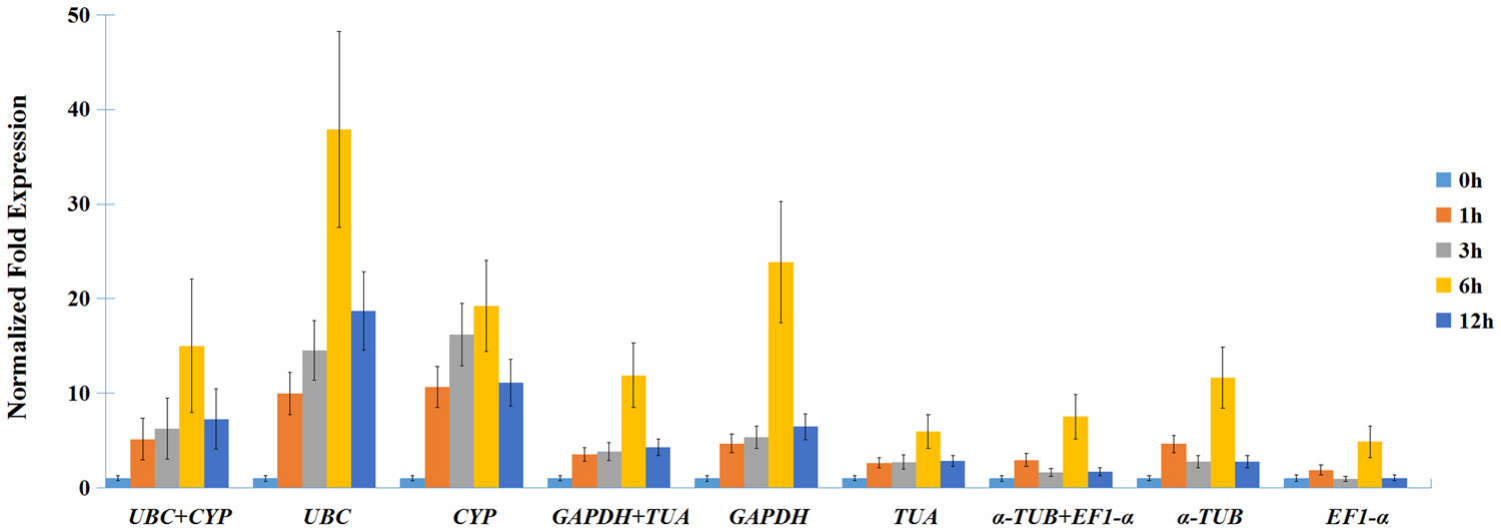
Relative quantification of *DkDREB2C* gene expression using validated reference genes for normalization under heat treatment in persimmon.

In *Arabidopsis*, *DREB2C* was induced by heat stress and might function as a late regulator [41,42]. To evaluate the reliability of the selected reference genes, *DkDREB2C* was cloned from persimmon, and the expression profiles of the *DkDREB2C* gene were calculated in persimmon under heat treatment by using the four most genes *CYP*, *GAPDH*, *TUA*, and *UBC* in individually and in group (Fig 5). The result showed that there were similar expression patterns: the expression levels of *DkDREB2C* gradually increased at 1 h, 3 h, and 6 h. In contrast, when using the least stable genes separately or a combination of *α-TUB* and *EF1-α* as reference genes for normalization, the expression patterns of *DkDREB2C* increased at 1 h, decreased at 3 h, and then increased again at 6 h (Fig 5).

## Discussion

In plant molecular biological research, RT-qPCR is used to quantify the gene expression levels due to its high sensitivity and specificity. Accurate measurement of gene expression with such a method relies on the stability of reference gene expression. However, the expression of commonly used reference genes could vary considerably in response to various factors [12,43]. This implies that the stability of the reference genes should be evaluated according to the test materials or experimental conditions. For persimmons, genomic information is limited in comparison to apple, pear, peach, citrus, and grape [2]. Thus, the 13 reference genes in our study were cloned based on transcriptome sequencing data (unpublished data).

In this study, we conducted a systematic evaluation of stability of 13 widely used genes under different abiotic stresses and hormone stimuli in persimmon. Before the implementation of RT-qPCR, we optimized the reaction system by performing standard curve analysis of five-fold diluted series (1, 5, 5^2^, 5^3^, 5^4^, and 5^5^ × dilution) to ensure the accuracy and repeatability of the subsequent test sample results. Using RT-qPCR, expression levels of 13 genes were determined as quantification cycle (Cq) values. Previous studies showed that the Cq values of reference genes within an acceptable range were regarded as reliable [17,20]. Here, most Cq values ranged from 16 to 30, which indicated that these genes could be used as candidate reference genes in this study.

To assess reference gene expression patterns more accurately, we performed two different statistical approaches (geNorm and NormFinder) [39,40]. Not surprisingly, the two programs gave slightly different results in the ranking of the most stably expressed candidate genes (S3 and S4 Tables), which may be due to the differences between the approaches [44,45]. For instance, under heat treatment, geNorm had the top four genes, in order, as *CYP*, *GAPDH*, *TUA*, and *UBC*, while NormFinder provided the opposite sorting. Under cold treatment, *RPII* and *TUA* were selected as the most stably expressed by geNorm, but were ranked sixth and fourth, respectively, by NormFinder. *α-TUB* emerged as the most stably expressed gene using NormFinder in the GA and ABA treatments, but it was ranked seventh and fifth by geNorm, respectively. However, both analysis programs produced the same unstable genes (*F-box*, *EF1-α*, *ACT*, and *RPL13*), which were ranked within the last five in all samples. In recent studies, *RPL13* performed poorly in peach [36], and the widely used persimmon reference gene *ACT* has been noted to show variable expression in soybean [46], *C*. *intermedia* [8], watermelon [19] and peach [36]. *F-box* and *EF1-α* were found to be unsuitable reference genes across all samples in oil palm [21], which is consistent with the results in our study, but in opposition to the results for cucumber [47]. In addition, *EF1-α* was unsuitable in melon [48], but suitable in wheat [49] and carrot [18]. Taken together, there is no complete unanimity of results with regards to the stabilities of these genes in different plant species, but it is clear that these genes should not be recommended as reference genes for normalization in persimmon. Altogether, these data suggested species-specific characteristics of reference genes [50].

When determining the best reference genes available for RT-qPCR normalization, we chose the common top-ranked genes from the two algorithms. Except for the "total" group, the optimal number of reference genes was the same for the remaining groups and each individual stress, as evidenced by the pairwise variation V_2/3_ value below the cut-off value (0.15). Based on the ranking orders, we could choose any two of the four genes (*CYP*, *GAPDH*, *TUA*, and *UBC*) as a suitable combination in the heat treatment, while any two of the three genes (*TUA*, *UBC*, and *CYP*) would be sufficient for cold stress. Similarly, *UBC* combined with *TUA* or *PP2A* would be marked as a suitable combination for NaCl stress; *RPL13* and *PP2A* were selected for GA stress; *TUA* and *SAND* were selected for ABA stress; and *UBC* combined with *CYP* or *α-TUB* would be suitable for SA stress. Similarly, we recommended *UBC* combined with *RPII* or *TUA* as the best combination under "abiotic stress", while *α-TUB* and *PP2A* were most stable reference genes in the "hormone stimuli". For "total", we recommended *UBC* and *GAPDH* as the best combination considering that the proposed 0.15 value is not a strict cut-off [39]. Our results indicated that no suitable genes can be used as references under a variety of conditions, as noted in other previous studies [8,17,18,21,36].

Among these stable reference genes, *UBC* displayed the maximum stability for almost all single stresses and groups in the present study, and good performance of *UBC* as a traditional reference gene has been shown in *Arabidopsis* [26]. Like *UBC*, *PP2A* performed well across most individual treatments, and good stability of *PP2A* was reported in other plants [8,17,19]. *GAPDH*, a classical housekeeping genes, had very poor expression stability in oil palm [21], peach [36], banana [51] and wheat [49]; on the contrary, its expression was stable in pepper [20] and carrot [18], which is consistent with the results of our study. Furthermore, *CYP* and *TUA* were unsuitable in cucumber [47]. *RPII* was the most suitable reference genes in peach [36]. These discrepancies about the most and least stable genes between different plant species emphasizes the importance of evaluating the expression stability of reference genes under specific experimental conditions and specific species.

Finally, further validation of the identified reference genes was performed by normalizing the relative expression of *DKDREB2C*. *DREB2C* is one of the *Arabidopsis* class 2 *DREBs* (dehydration responsive element binding), which plays a critical role in improving plant resistance [52]. Its expression pattern under heat stress differed from *DREB2A* that was rapidly and transiently induced and peaked within 1 h [53]. In our study, when the four most stable genes were used separately or any two of them as a group, the transcript level of *DkDREB2C* increased with the extension of treatment time, peaked at 6 h, and decreased between 6 h and 12 h. This was slightly different from *Arabidopsis*, in which its expression was observed at 3 h after heat treatment, and then continued to increase for 12 h or 24 h [41,42]. We suspect that this might be due to different experimental materials. When using the least stable genes separately or in combination, we found obvious differences. The transcript level of *DkDREB2C* peaked at 1 h and 6 h. These results further suggested that *CYP*, *GAPDH*, *TUA*, and *UBC* are suitable for RT-qPCR under heat treatment in persimmon, while *α-TUB* and *EF1-α* were not suitable. With this validation, we found that an inappropriate reference gene might affect the normalization results.

## Conclusions

In conclusion, 13 candidate reference genes were first evaluated under different treatments (heat, cold, and salt, GA, SA, and ABA) in persimmon. As a result of the analysis, we identified suitable persimmon reference genes under specific experimental conditions. Among them, *UBC* and *GAPDH* could be considered the most suitable reference genes across all samples, whereas the commonly used persimmon reference gene *ACT* as well as genes *F-box*, *EF1-α*, and *RPL13* could not be recommended as an optimal reference gene for normalization of persimmon gene expression data. We concluded that the use of an unsuitable reference gene might yield misleading results. In other words, it is essential to choose proper reference genes for RT-qPCR analysis to obtain accurate and reliable data depending on both the test materials and experimental conditions.

## Supporting Information

S1 Fig Amplification specificity of the 13 reference genes in qPCR. Amplification fragments were separated by 1.5% agarose gel electrophoresis.
(TIF)

S2 Fig Standard curves of the 13 candidate reference genes. The amplification efficiency (E) of each reference gene was calculated from the standard curve with the following equation: E = [10^(-1/slope)^-1]×100%. C0 represents the initial copy number of cDNA.
(TIF)

S3 Fig Nucleic acid and deduced amino acid sequences of *ACT* from persimmon. (TIF)

S4 Fig Nucleic acid and deduced amino acid sequences of *α-TUB* from persimmon. (TIF)

S5 Fig Nucleic acid and deduced amino acid sequences of *β-TUB* from persimmon. (TIF)

S6 Fig Nucleic acid and deduced amino acid sequences of *UBC* from persimmon. (TIF)

S7 Fig Nucleic acid and deduced amino acid sequences of *CYP* from persimmon. (TIF)

S8 Fig Nucleic acid and deduced amino acid sequences of *RPL13* from persimmon. (TIF)

S9 Fig Nucleic acid and deduced amino acid sequences of *PP2A* from persimmon. (TIF)

S10 Fig Nucleic acid and deduced amino acid sequences of *GAPDH* from persimmon. (TIF)

S11 Fig Nucleic acid and deduced amino acid sequences of *EF1-α* from persimmon. (TIF)

S12 Fig Nucleic acid and deduced amino acid sequences of *F-box* from persimmon. (TIF)

S13 Fig Nucleic acid and deduced amino acid sequences of *RPII* from persimmon. (TIF)

S14 Fig Nucleic acid and deduced amino acid sequences of *TUA* from persimmon. (TIF)

S15 Fig Nucleic acid and deduced amino acid sequences of *SAND* from persimmon. (TIF)

S1 Table Primer sequences of reference genes and *DkDREB2C* in persimmon (clone). (DOCX)

S2 Table Raw Cq values in persimmon. Plants were submitted to the following treatments: heat, cold, salt, salicylic acid (SA), gibberellins (GA), and abscisic acid (ABA); CK: samples without any treatment.
(DOCX)

S3 Table Gene expression stability under multiple stresses ranked by geNorm and NormFinder. (DOCX)

S4 Table Gene expression stability under individual stress ranked by geNorm and NormFinder. (DOCX)
