# Supplementary figures and images for: Selection of Suitable Reference Genes for RT-qPCR Normalization under Abiotic Stresses and Hormone Stimulation in Persimmon (Diospyros kaki Thunb)
Source: PLoS One. 2016 Aug 11;11(8):e0160885. doi: 10.1371/journal.pone.0160885 (PMC4981405; doi:10.1371/journal.pone.0160885)

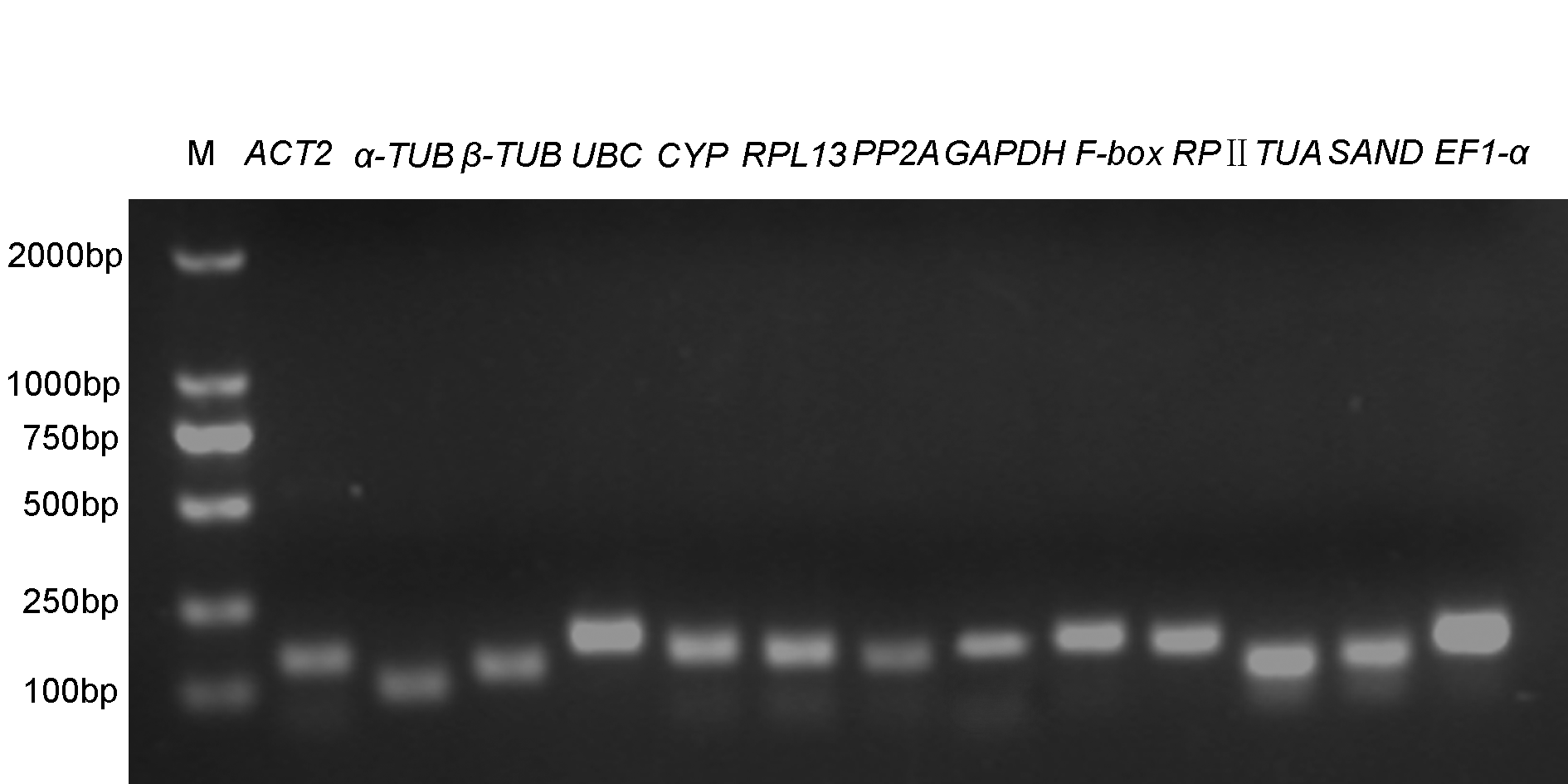

Supplement: S1 Fig — Amplification fragments were separated by 1.5% agarose gel electrophoresis. (TIF) [file pone.0160885.s001.tif]

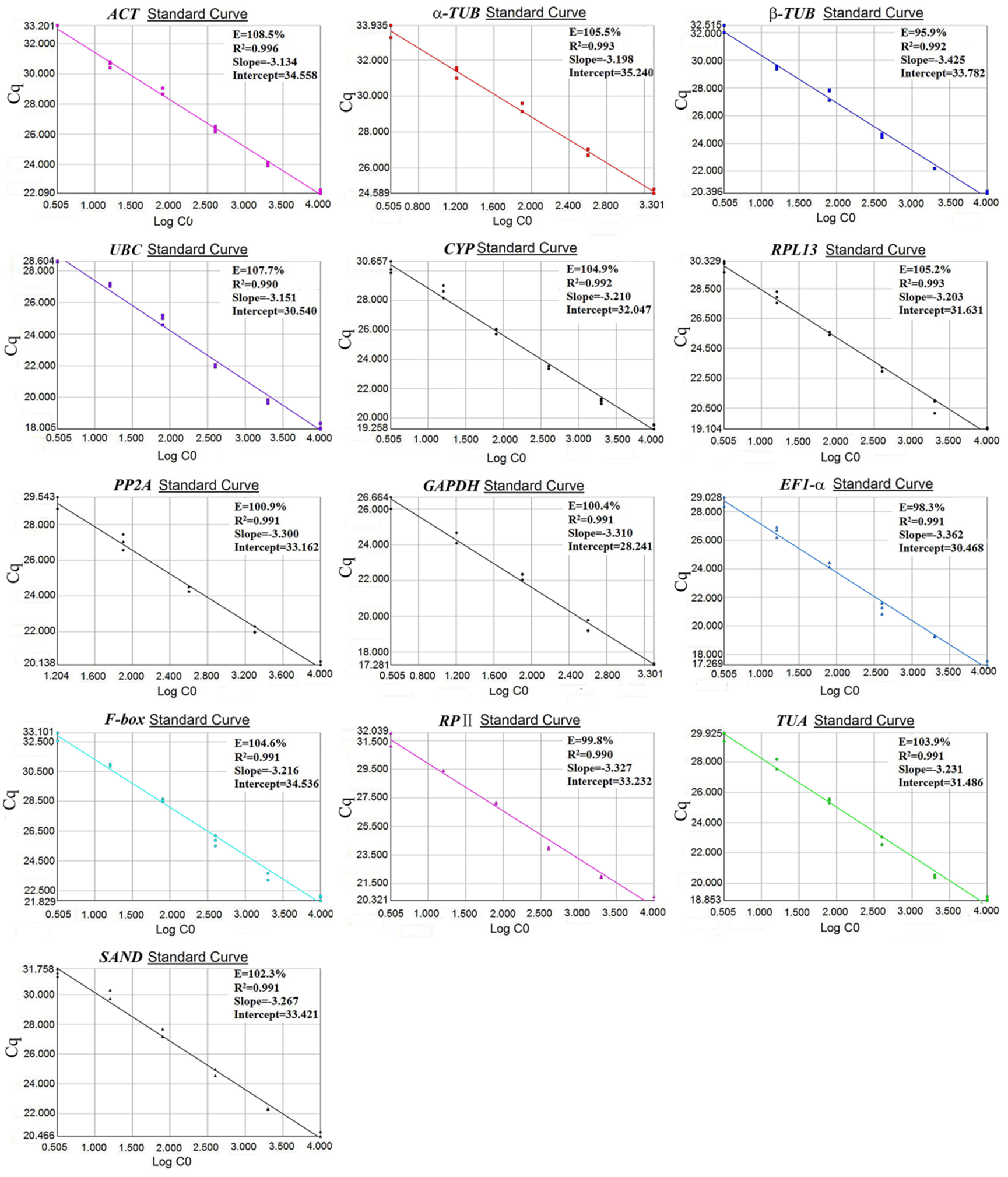

Supplement: S2 Fig — The amplification efficiency (E) of each reference gene was calculated from the standard curve with the following equation: E = [10(-1/slope)-1]×100%. C0 represents the initial copy number of cDNA. (TIF) [file pone.0160885.s002.tif]

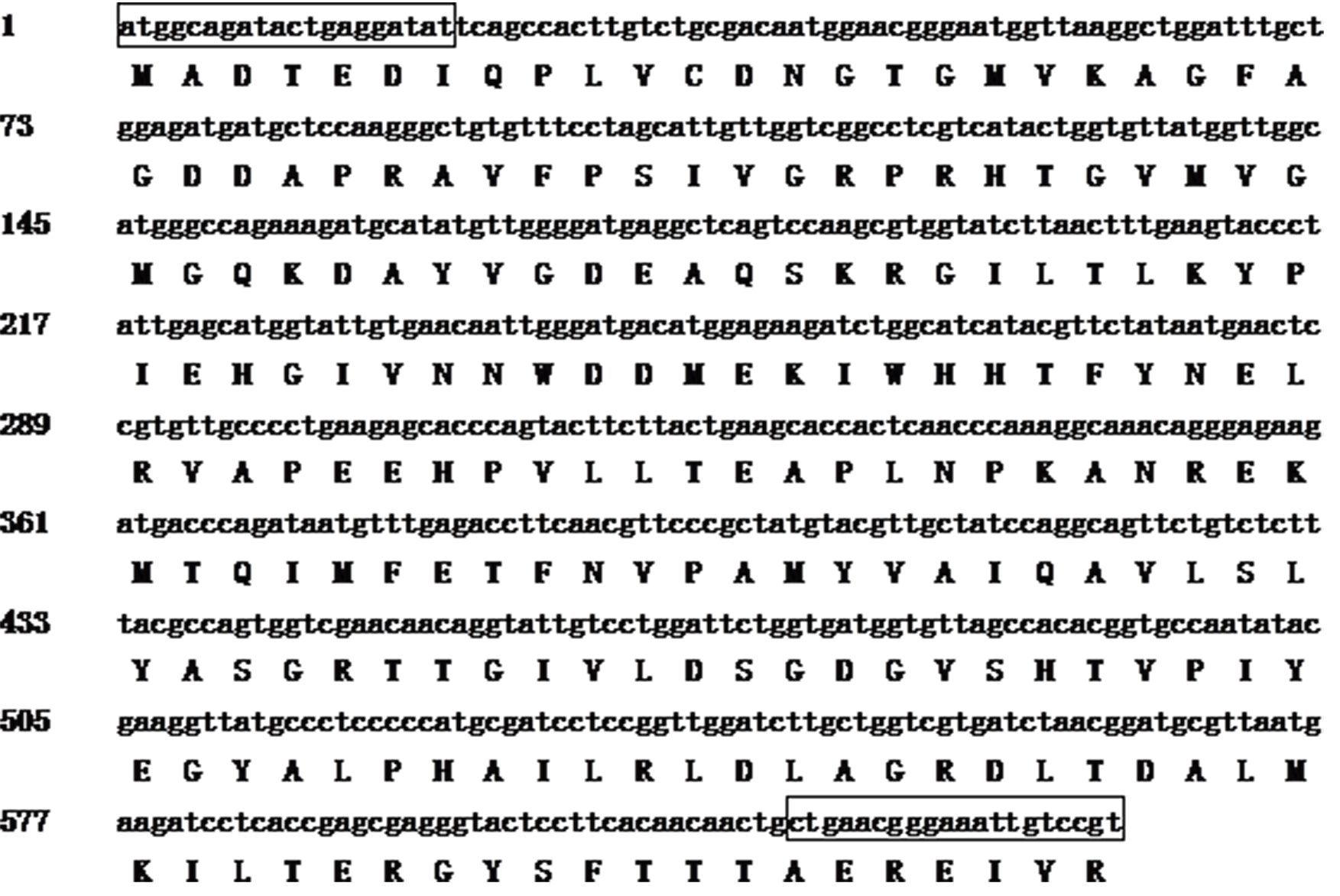

Supplement: S3 Fig — (TIF) [file pone.0160885.s003.tif]

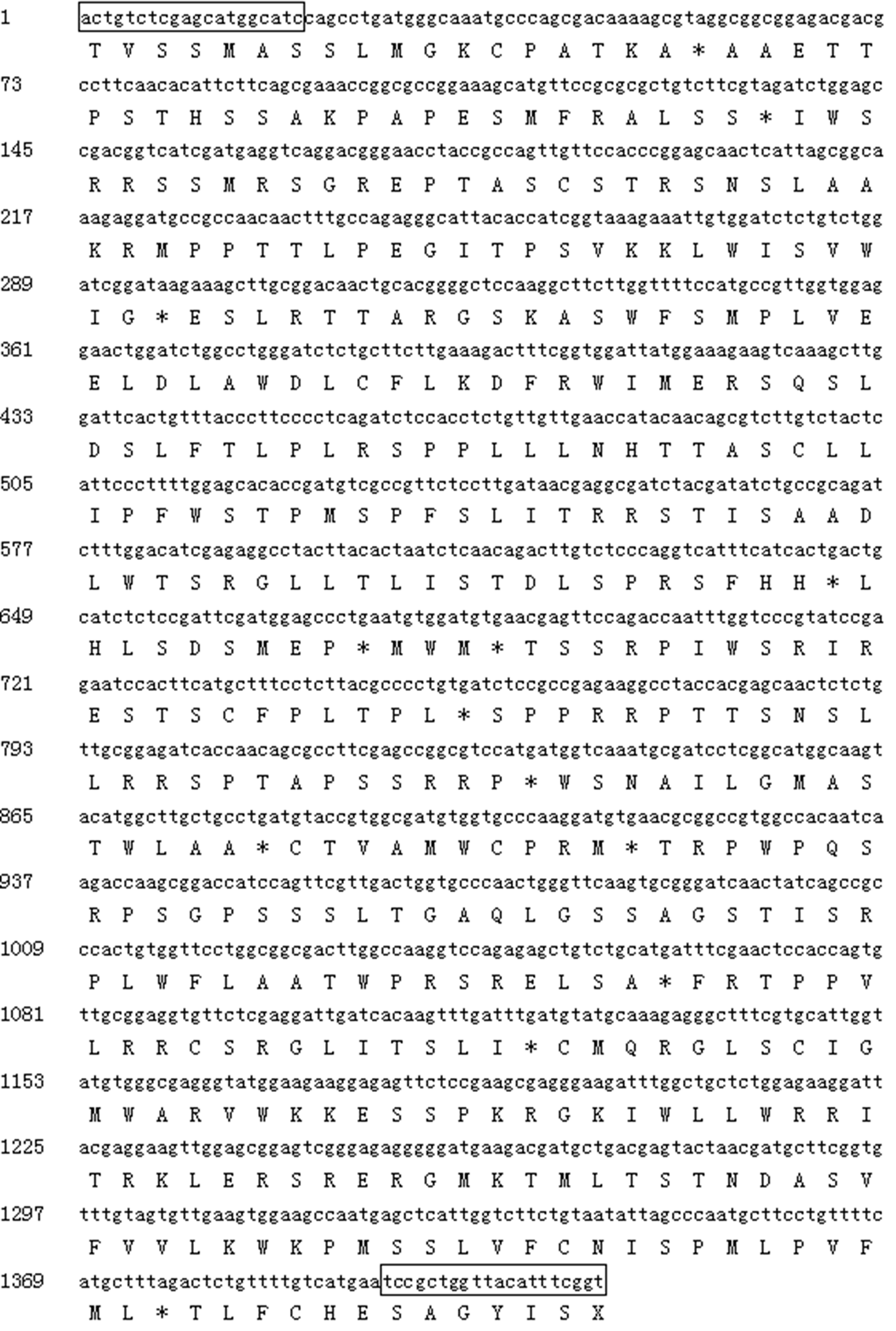

Supplement: S4 Fig — (TIF) [file pone.0160885.s004.tif]

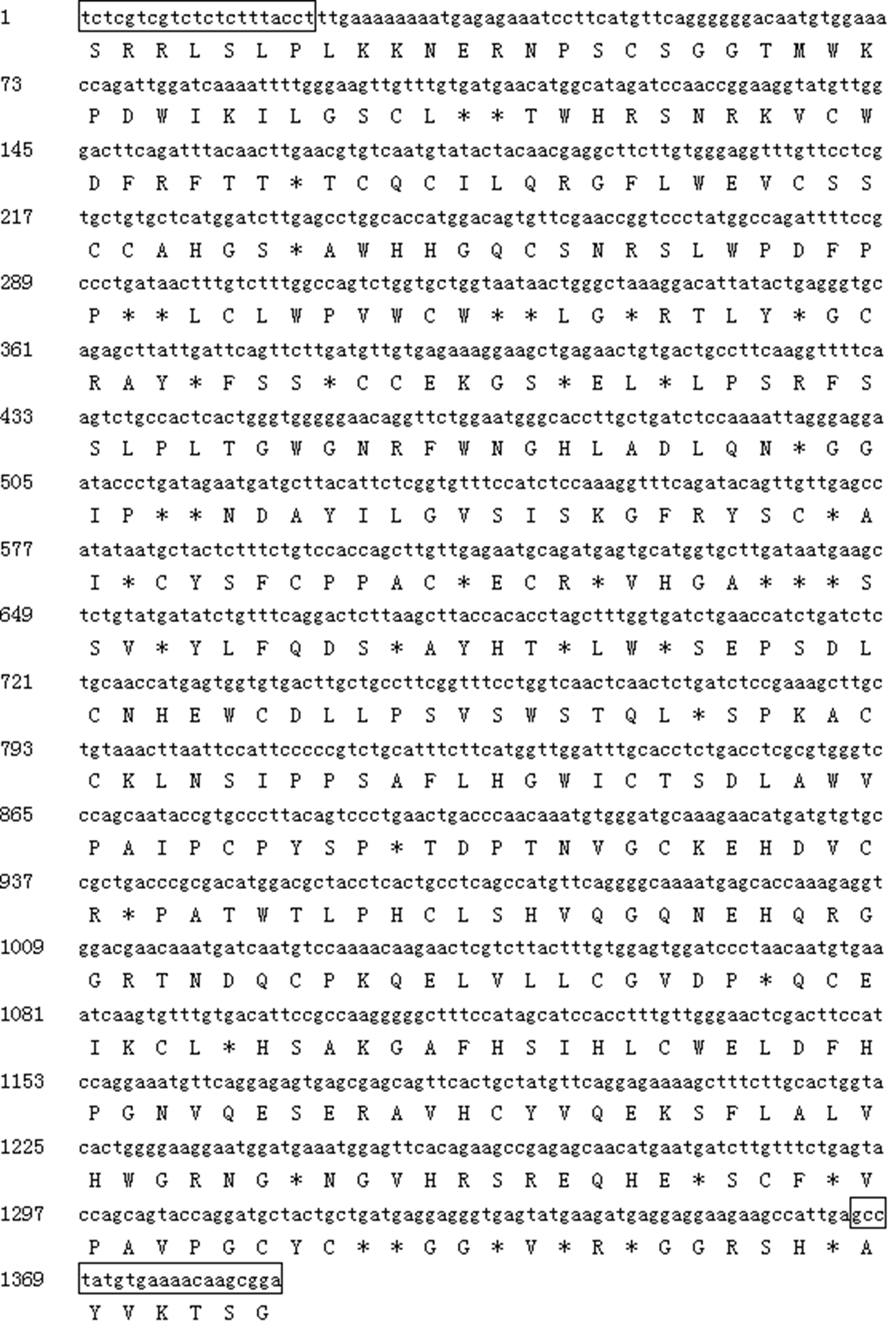

Supplement: S5 Fig — (TIF) [file pone.0160885.s005.tif]

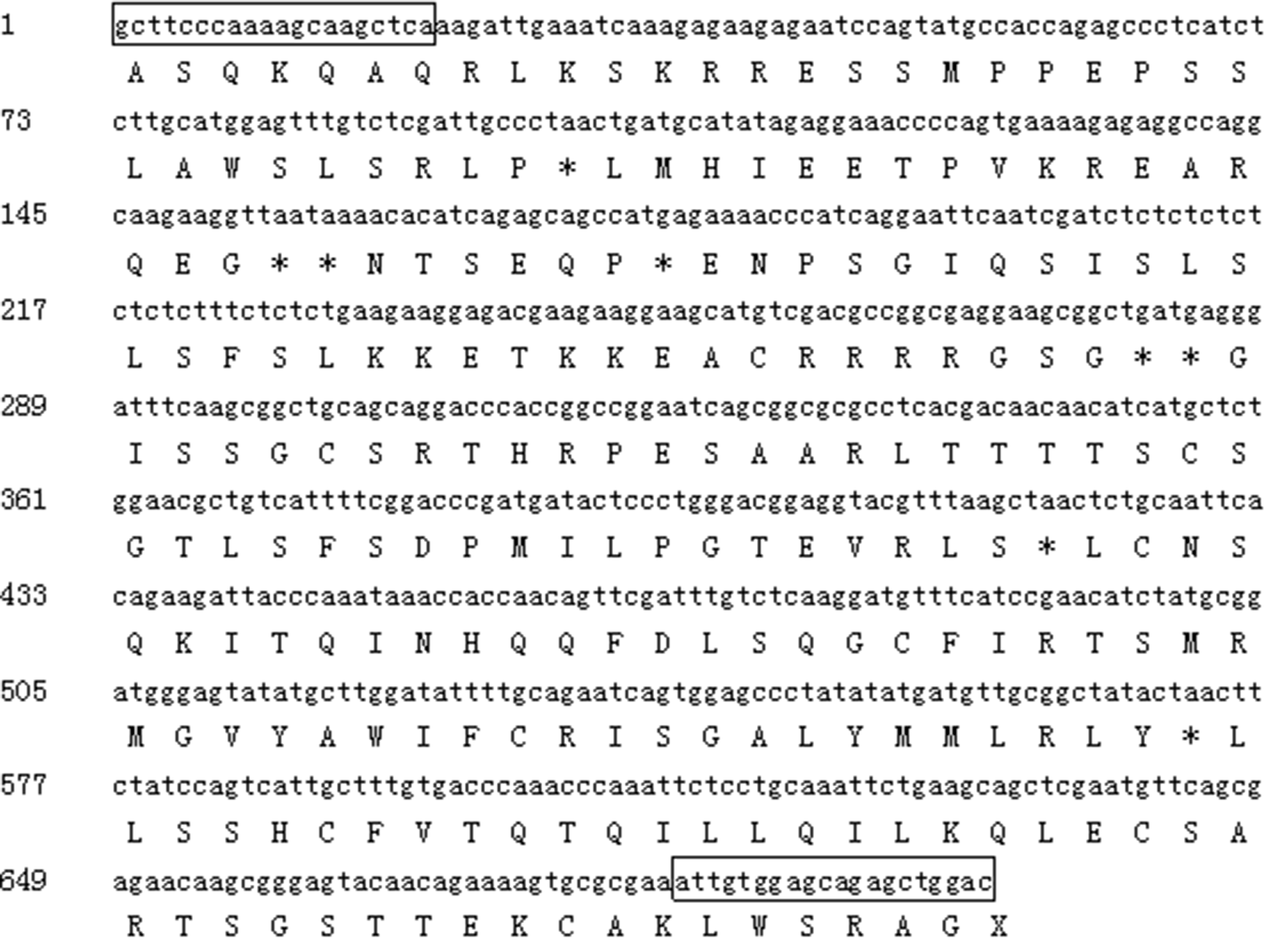

Supplement: S6 Fig — (TIF) [file pone.0160885.s006.tif]

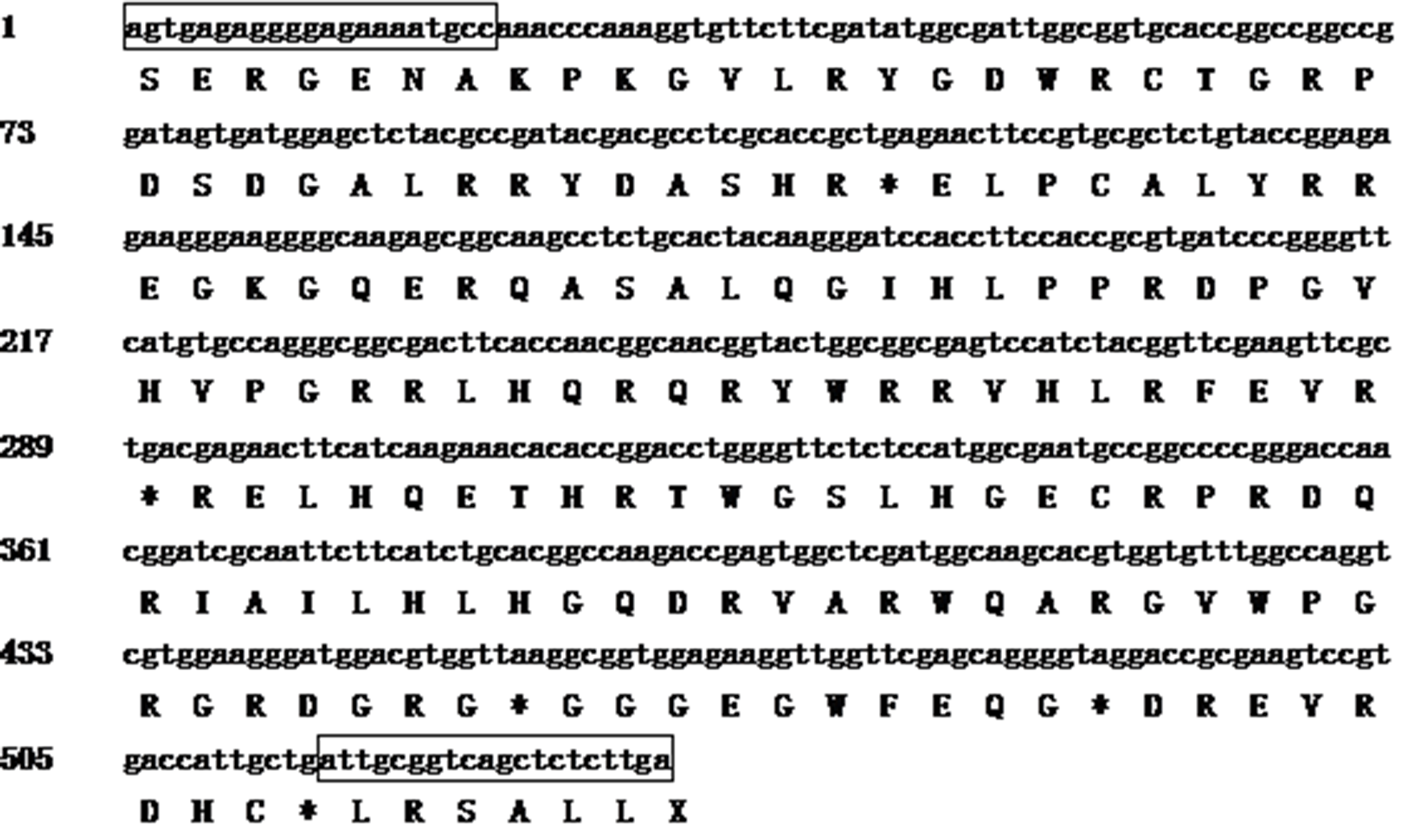

Supplement: S7 Fig — (TIF) [file pone.0160885.s007.tif]

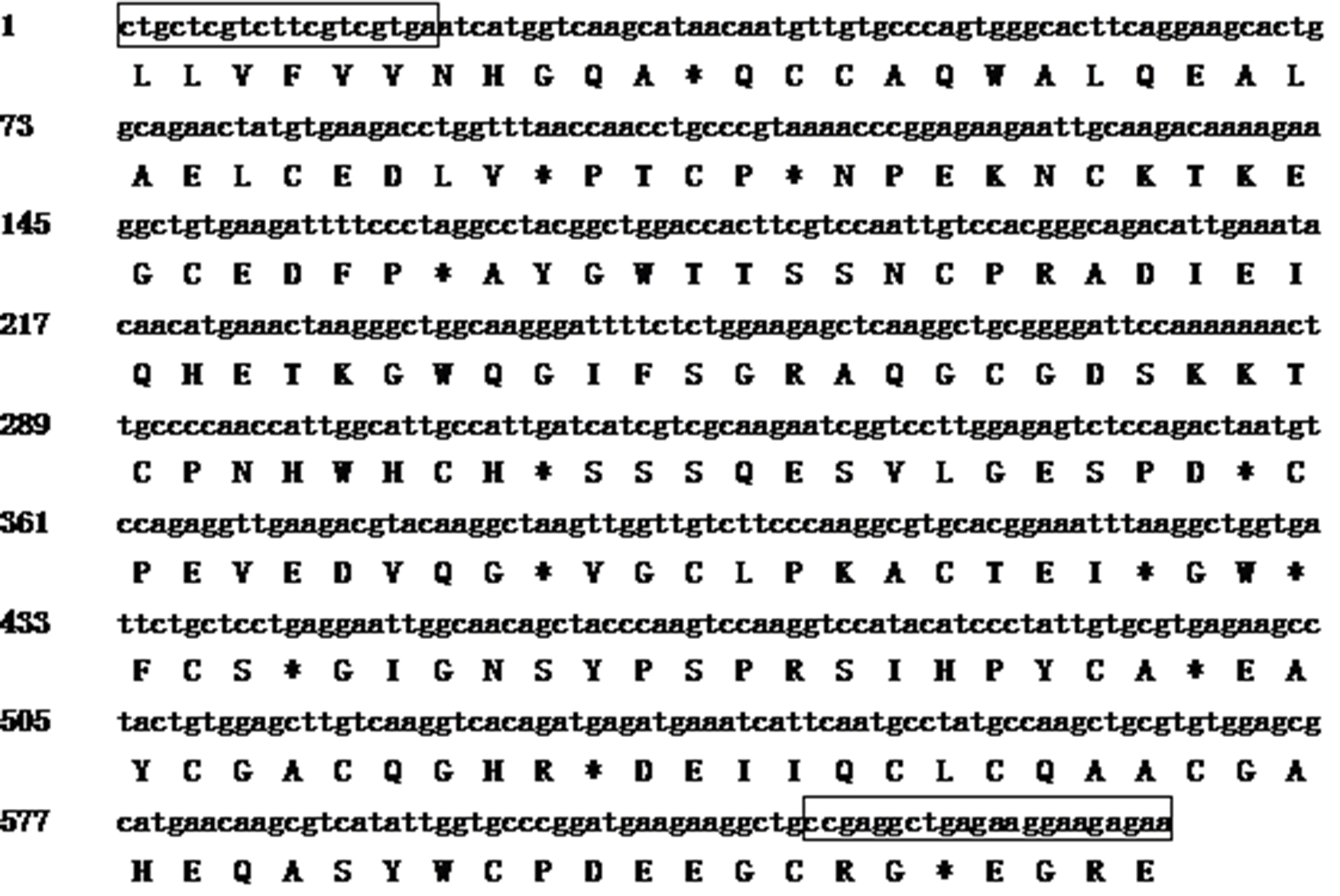

Supplement: S8 Fig — (TIF) [file pone.0160885.s008.tif]

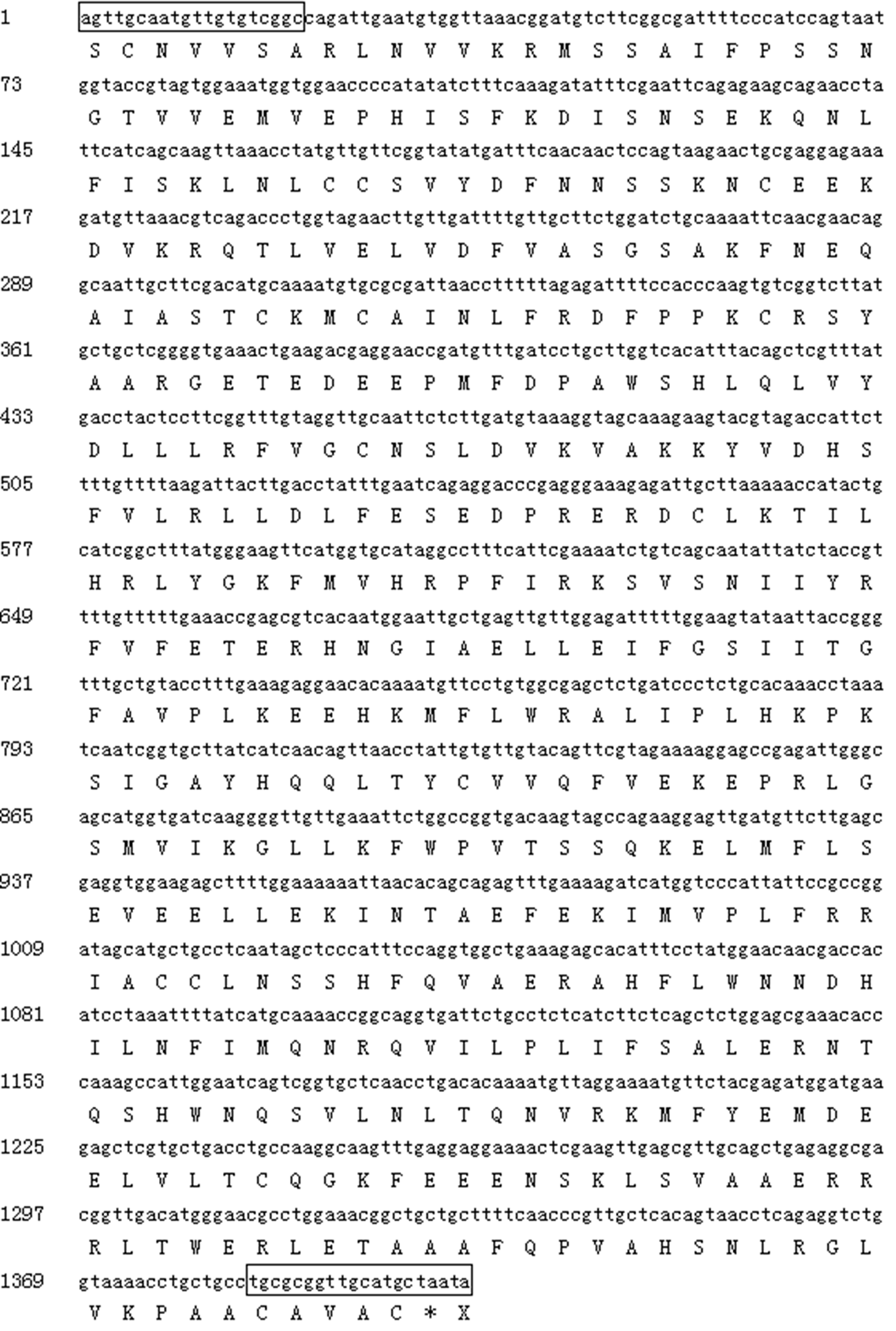

Supplement: S9 Fig — (TIF) [file pone.0160885.s009.tif]

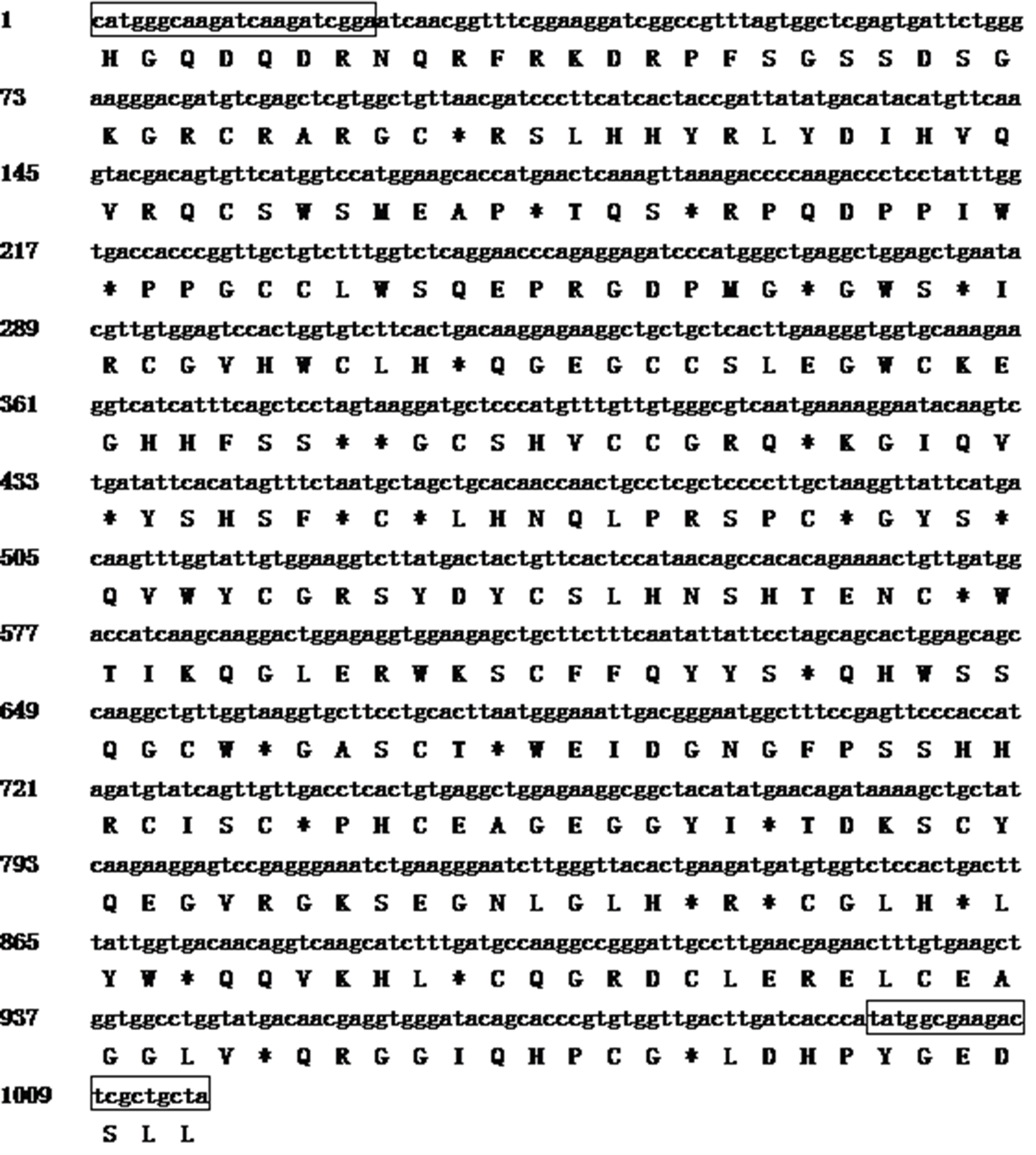

Supplement: S10 Fig — (TIF) [file pone.0160885.s010.tif]

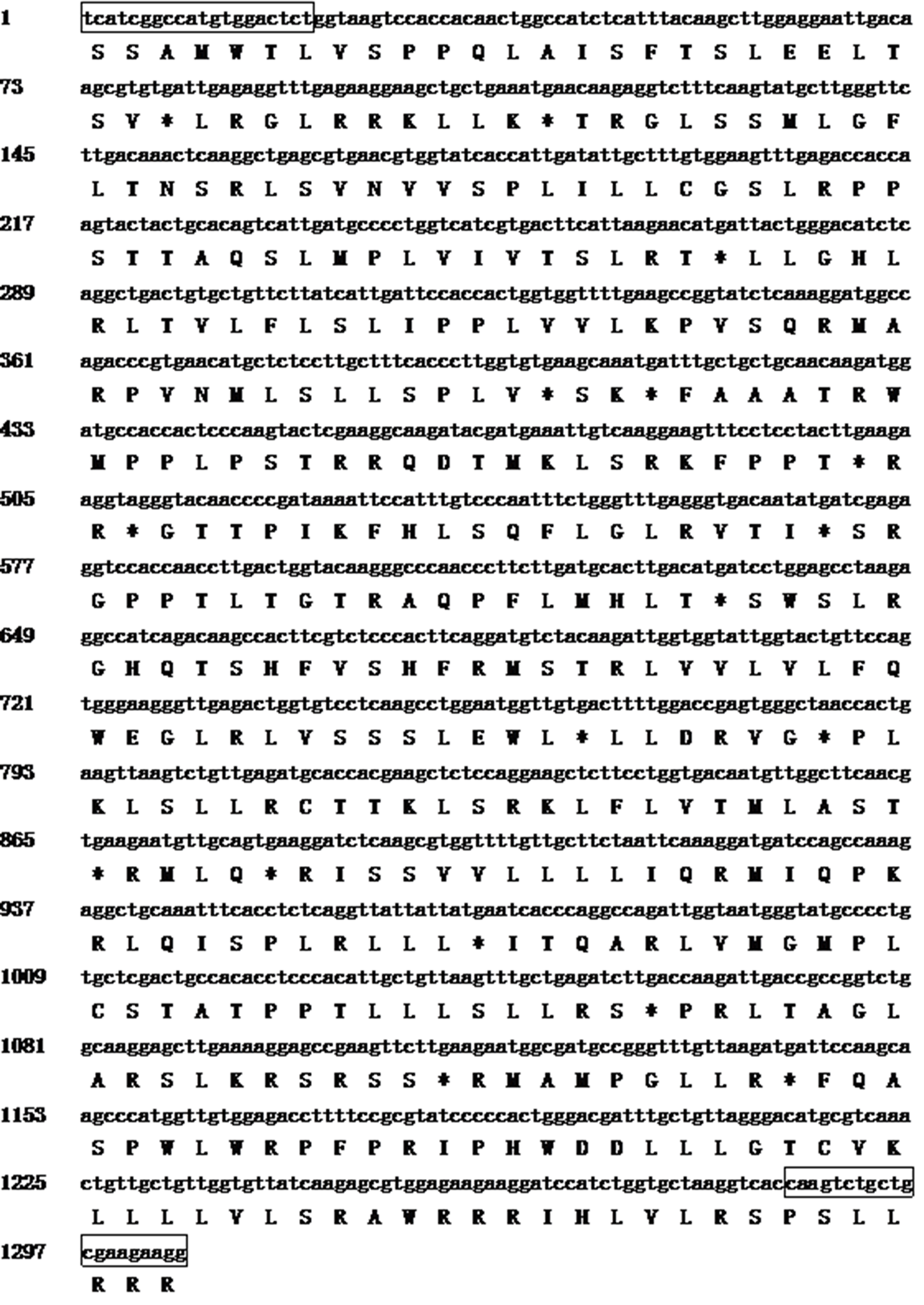

Supplement: S11 Fig — (TIF) [file pone.0160885.s011.tif]

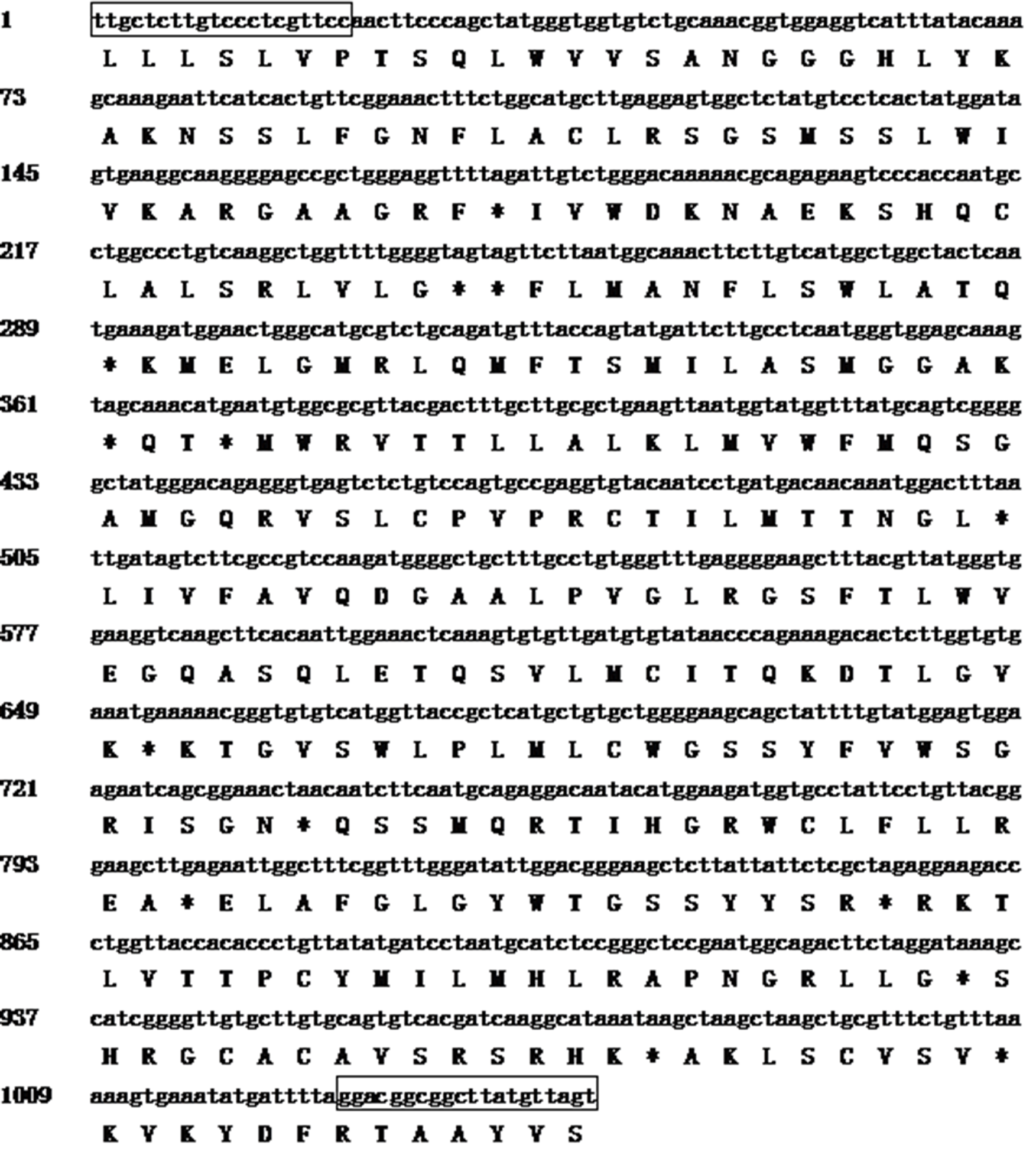

Supplement: S12 Fig — (TIF) [file pone.0160885.s012.tif]

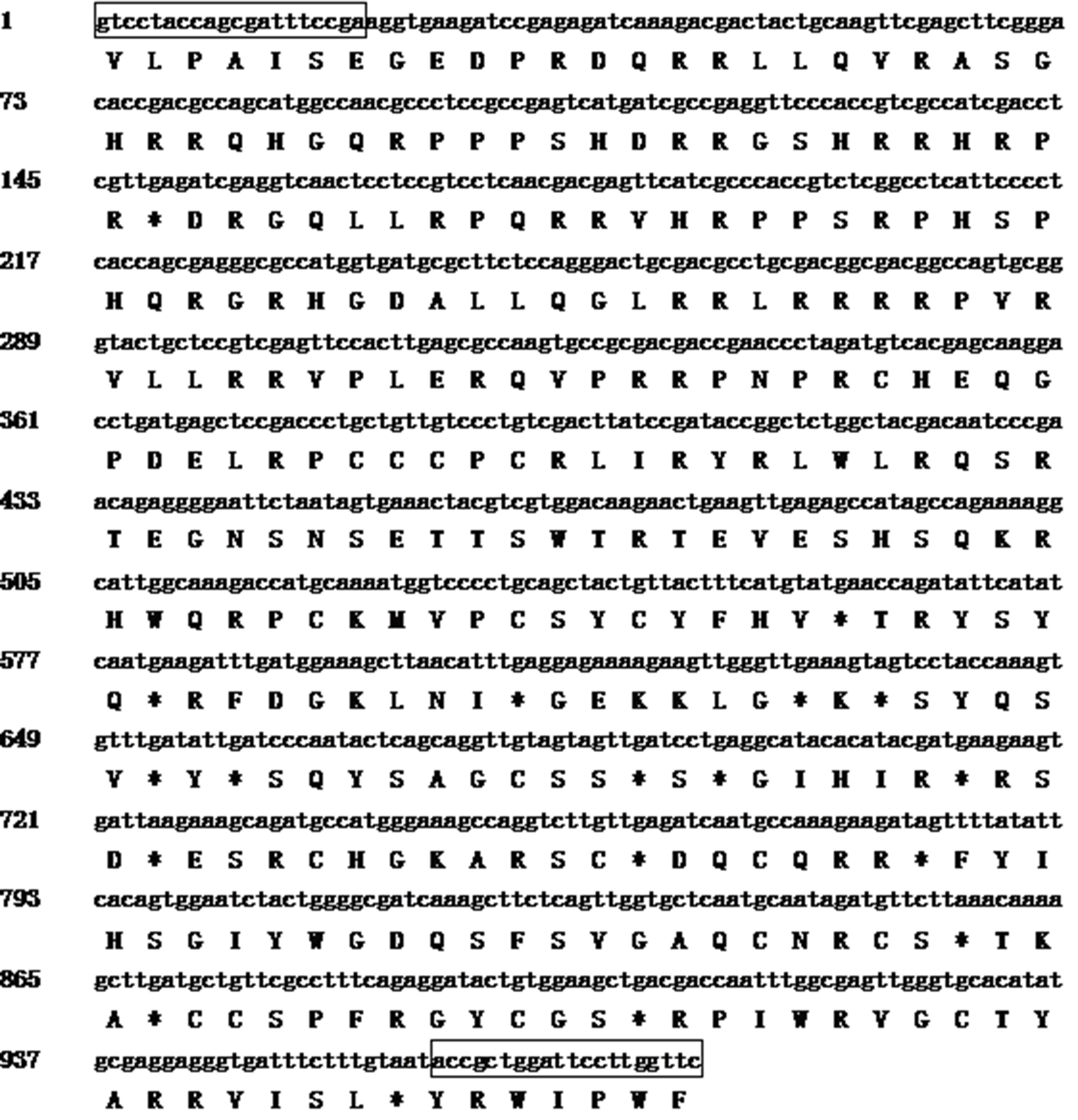

Supplement: S13 Fig — (TIF) [file pone.0160885.s013.tif]

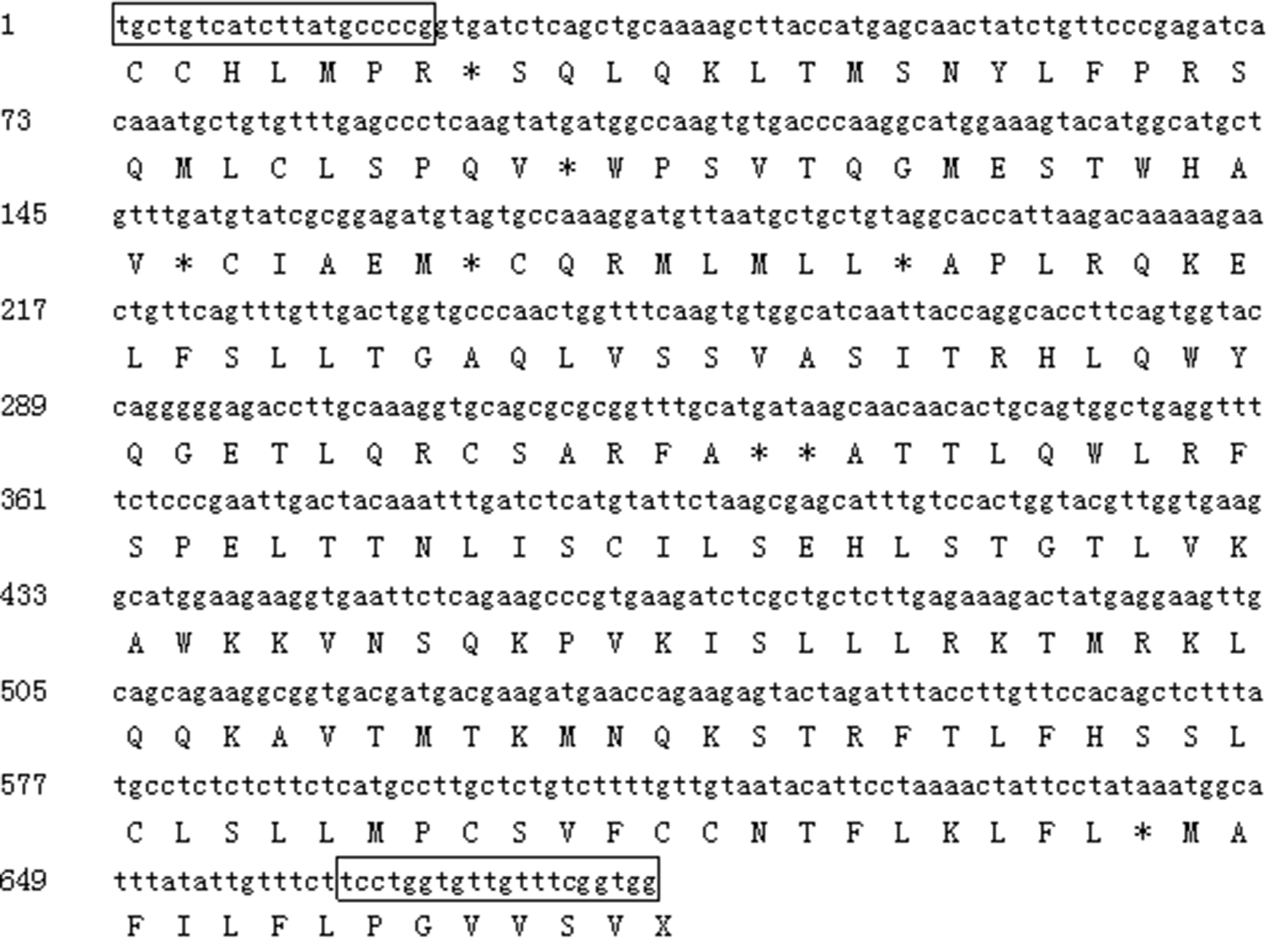

Supplement: S14 Fig — (TIF) [file pone.0160885.s014.tif]

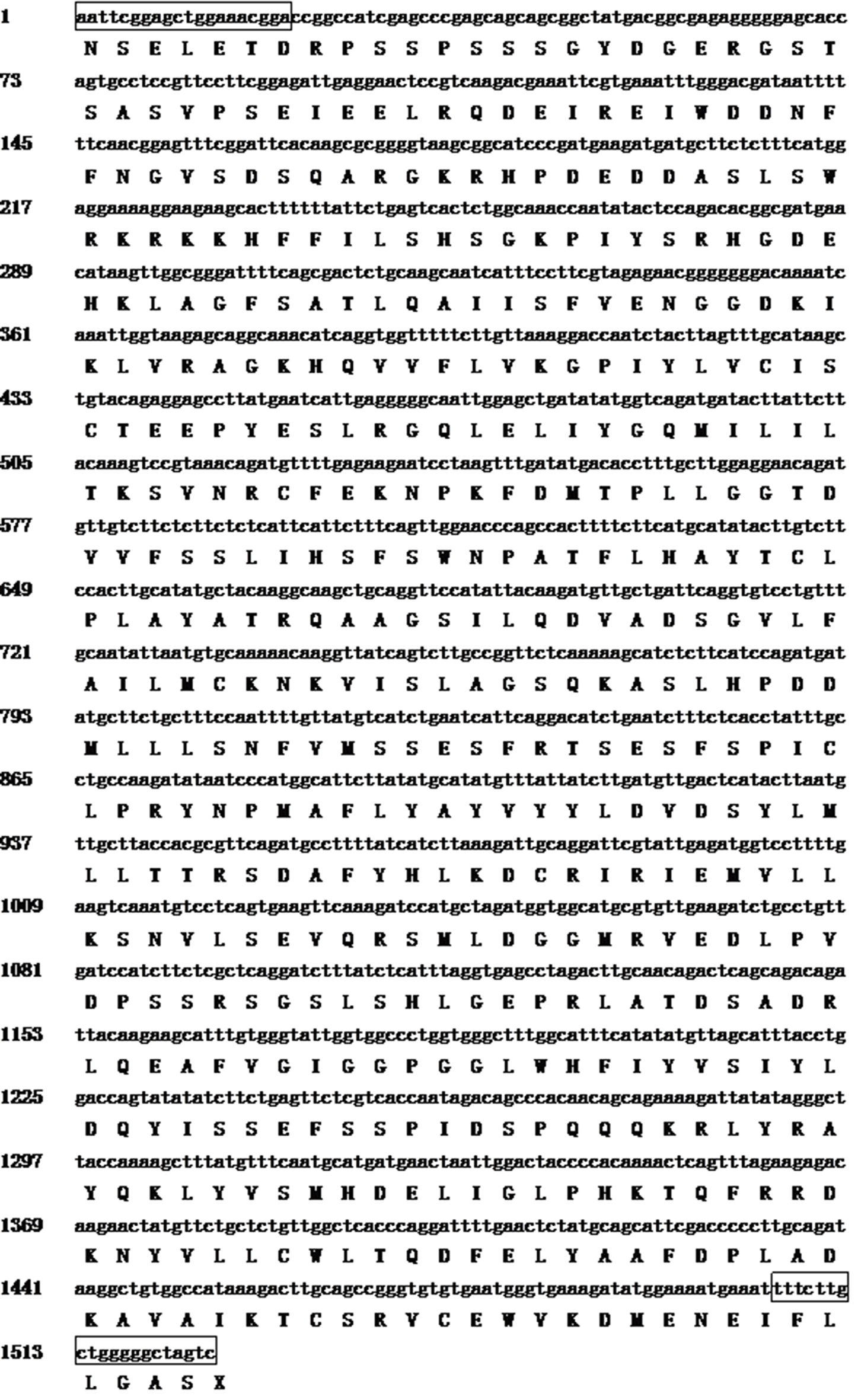

Supplement: S15 Fig — (TIF) [file pone.0160885.s015.tif]
