# Supplementary material for: Selection of Suitable Reference Genes for RT-qPCR Normalization under Abiotic Stresses and Hormone Stimulation in Persimmon (Diospyros kaki Thunb)
Source: PLoS One. 2016 Aug 11;11(8):e0160885. doi: 10.1371/journal.pone.0160885 (PMC4981405; doi:10.1371/journal.pone.0160885)
Supplement: S1 Table — (DOCX) [file pone.0160885.s016.docx]

**Table S1.** Primer sequences of reference genes and *DkDREB2C* in persimmon ( clone).

| **Gene symbol** | **Primer sequence (5ʹ–3ʹ) forward/ reverse** | **Amplicon length (bp)** |
| --- | --- | --- |
| *ACT* | ATGGCAGATACTGAGGATAT/  ACGGACAATTTCCCGTTCAG | 640 |
| *α-TUB* | ACTGTCTCGAGCATGGCATC/  ACCGAAATGTAACCAGCGGA | 1415 |
| *β-TUB* | TCTCGTCGTCTCTCTTTACCT/  TCCGCTTGTTTTCACATAGGC | 1386 |
| *UBC* | GCTTCCCAAAAGCAAGCTCA/  GTCCAGCTCTGCTCCACAAT | 703 |
| *CYP* | AGTGAGAGGGGAGAAAATGCC/  TCAAGAGAGCTGACCGCAAT | 535 |
| *RPL13* | CTGCTCGTCTTCGTCGTGA/  TTCTCTTCCTTCTCAGCCTCGG | 639 |
| *PP2A* | AGTTGCAATGTTGTGTCGGC/  TATTAGCATGCAACCGCGCA | 1403 |
| *GAPDH* | CATGGGCAAGATCAAGATCGGA/  TAGCAGCGAGTCTTCGCCATA | 1017 |
| *EF 1-α* | TCATCGGCCATGTGGACTCT/  CCTTCTTCGCAGCAGACTTG | 1305 |
| *F-box* | TTGCTCTTGTCCCTCGTTCC/  ACTAACATAAGCCGCCGTCC | 1047 |
| *RPII* | GTCCTACCAGCGATTTCCGA/  GAACCAAGGAATCCAGCGGT | 981 |
| *TUA* | TGCTGTCATCTTATGCCCCG/  CCACCGAAACAACACCAGGA | 682 |
| *SAND* | AATTCGGAGCTGGAAACGGA/  GACTAGCCCCCAGCAAGAAA | 1525 |
| *DkDREB2C* | ACGTAGACGCAAGCTTTGGA/  AAAGGGCCATCCAGCTACAC | 1468 |
