# Supplementary material for: Selection of Suitable Reference Genes for RT-qPCR Normalization under Abiotic Stresses and Hormone Stimulation in Persimmon (Diospyros kaki Thunb)
Source: PLoS One. 2016 Aug 11;11(8):e0160885. doi: 10.1371/journal.pone.0160885 (PMC4981405; doi:10.1371/journal.pone.0160885)
Supplement: S2 Table — Plants were submitted to the following treatments: heat, cold, salt, salicylic acid (SA), gibberellins (GA), and abscisic acid (ABA); CK: samples without any treatment. (DOCX) [file pone.0160885.s017.docx]

**Table S2.** Raw Cq values in persimmon.

Plants were submitted to the following treatments: heat, cold, salt, salicylic acid (SA), gibberellins (GA), and abscisic acid (ABA); CK: samples without any treatment.

|  | *ACT* | *α-TUB* | *β-TUB* | *UBC* | *CYP* | *RPL13* | *PP2A* | *GAPDH* | *EF1-α* | *F-box* | *RPII* | *TUA* | *SAND* |
| --- | --- | --- | --- | --- | --- | --- | --- | --- | --- | --- | --- | --- | --- |
| CK-1 | 24.96 | 22.17 | 20.81 | 18.97 | 21.13 | 20.00 | 23.51 | 18.21 | 19.33 | 23.27 | 22.19 | 20.63 | 22.94 |
| CK-2 | 24.90 | 22.19 | 21.06 | 19.12 | 21.52 | 20.28 | 24.01 | 18.40 | 19.33 | 23.11 | 22.13 | 20.63 | 22.55 |
| CK-3 | 25.23 | 22.89 | 21.18 | 19.24 | 21.67 | 20.99 | 24.39 | 19.03 | 19.62 | 23.36 | 21.91 | 20.64 | 23.11 |
| Hea t-1 | 23.97 | 23.35 | 19.76 | 18.95 | 20.87 | 20.12 | 24.27 | 18.33 | 19.96 | 22.07 | 21.24 | 20.43 | 22.18 |
| Hea t-2 | 23.37 | 23.34 | 19.74 | 18.73 | 20.61 | 19.78 | 23.99 | 17.88 | 20.49 | 22.15 | 21.02 | 19.80 | 21.89 |
| Hea t-3 | 23.71 | 22.93 | 19.42 | 18.48 | 20.33 | 19.32 | 23.50 | 17.58 | 20.53 | 22.08 | 20.86 | 19.55 | 21.42 |
| Hea t-4 | 23.21 | 22.82 | 19.16 | 18.35 | 20.19 | 19.32 | 23.39 | 17.45 | 19.64 | 21.71 | 21.18 | 19.73 | 21.67 |
| Hea t-5 | 23.51 | 22.77 | 19.36 | 18.13 | 19.95 | 19.11 | 23.24 | 17.23 | 19.96 | 22.08 | 20.66 | 19.60 | 21.78 |
| Hea t-6 | 24.67 | 22.78 | 18.92 | 18.36 | 20.02 | 18.97 | 23.26 | 17.29 | 19.95 | 22.08 | 20.82 | 19.21 | 21.36 |
| Hea t-7 | 23.07 | 22.57 | 19.16 | 18.15 | 19.85 | 19.00 | 23.14 | 17.17 | 19.17 | 21.54 | 20.82 | 19.28 | 20.96 |
| Hea t-8 | 23.34 | 22.52 | 19.12 | 18.07 | 19.87 | 19.14 | 23.08 | 17.22 | 19.18 | 22.17 | 20.56 | 19.34 | 21.12 |
| Hea t-9 | 23.18 | 22.63 | 19.33 | 18.10 | 20.47 | 18.61 | 23.38 | 17.38 | 19.27 | 22.74 | 20.59 | 19.89 | 21.33 |
| Cold-1 | 24.82 | 24.23 | 21.15 | 19.40 | 21.29 | 19.20 | 24.50 | 18.84 | 22.02 | 25.36 | 21.72 | 20.43 | 23.11 |
| Cold-2 | 24.46 | 23.66 | 21.10 | 19.21 | 21.10 | 18.97 | 24.17 | 18.29 | 22.22 | 25.47 | 21.61 | 20.22 | 22.50 |
| Cold-3 | 24.31 | 24.12 | 21.09 | 19.10 | 20.78 | 18.49 | 23.89 | 18.08 | 22.57 | 25.38 | 21.43 | 20.09 | 22.22 |
| Cold-4 | 23.17 | 22.33 | 19.63 | 19.00 | 20.54 | 18.50 | 22.60 | 17.44 | 20.21 | 23.47 | 21.41 | 19.63 | 21.85 |
| Cold-5 | 22.76 | 22.60 | 19.69 | 18.68 | 20.25 | 18.33 | 22.43 | 17.31 | 20.41 | 23.96 | 21.26 | 19.41 | 21.56 |
| Cold-6 | 24.26 | 22.49 | 19.20 | 18.73 | 20.31 | 18.29 | 22.36 | 17.32 | 20.54 | 23.93 | 21.42 | 19.94 | 21.28 |
| Cold-7 | 23.35 | 22.90 | 20.76 | 19.34 | 21.30 | 19.00 | 23.02 | 18.04 | 20.35 | 24.16 | 21.94 | 20.19 | 22.19 |
| Cold-8 | 23.42 | 22.52 | 20.25 | 19.24 | 21.02 | 19.29 | 22.99 | 18.08 | 20.31 | 24.58 | 21.87 | 20.06 | 22.45 |
| Cold-9 | 23.46 | 22.69 | 19.93 | 19.37 | 21.71 | 18.56 | 23.10 | 18.26 | 20.34 | 25.21 | 22.06 | 20.49 | 21.48 |
| NaCl-1 | 30.21 | 28.59 | 25.07 | 23.57 | 24.52 | 23.20 | 28.65 | 22.62 | 26.85 | 30.90 | 26.57 | 25.59 | 27.51 |
| NaCl-2 | 29.30 | 28.11 | 24.77 | 23.26 | 24.20 | 22.95 | 28.72 | 22.10 | 26.69 | 31.39 | 26.19 | 25.09 | 27.88 |
| NaCl-3 | 30.46 | 28.05 | 24.67 | 23.25 | 24.18 | 22.51 | 28.47 | 21.85 | 27.22 | 31.70 | 26.31 | 24.73 | 27.92 |
| NaCl-4 | 31.55 | 28.31 | 26.26 | 23.61 | 24.22 | 22.92 | 29.21 | 22.30 | 26.17 | 31.50 | 26.06 | 25.03 | 28.01 |
| NaCl-5 | 29.94 | 28.55 | 26.38 | 23.48 | 24.08 | 22.44 | 28.85 | 22.12 | 26.57 | 31.06 | 25.84 | 25.09 | 28.09 |
| NaCl-6 | 31.35 | 28.26 | 25.47 | 23.51 | 23.94 | 22.27 | 28.86 | 22.02 | 26.38 | 30.54 | 26.43 | 24.88 | 27.33 |
| NaCl-7 | 29.28 | 27.59 | 24.81 | 22.89 | 23.52 | 22.09 | 28.13 | 21.29 | 25.49 | 30.95 | 25.93 | 24.58 | 27.22 |
| NaCl-8 | 29.68 | 27.34 | 26.08 | 22.91 | 23.41 | 22.21 | 28.41 | 21.39 | 25.69 | 30.40 | 26.08 | 24.48 | 27.58 |
| NaCl-9 | 29.27 | 27.13 | 23.60 | 22.92 | 23.89 | 22.74 | 28.13 | 21.49 | 25.91 | 31.24 | 26.15 | 24.64 | 26.26 |
| GA-1 | 24.94 | 23.02 | 21.05 | 19.39 | 21.13 | 20.60 | 24.31 | 18.64 | 21.23 | 23.97 | 23.30 | 21.81 | 23.89 |
| GA-2 | 24.18 | 22.56 | 20.95 | 19.14 | 20.90 | 20.31 | 23.99 | 18.19 | 21.47 | 24.00 | 22.88 | 21.42 | 23.78 |
| GA-3 | 23.94 | 22.23 | 20.80 | 20.39 | 20.58 | 19.91 | 23.55 | 18.02 | 21.84 | 24.03 | 22.80 | 21.28 | 23.69 |
| GA-4 | 23.90 | 22.40 | 20.68 | 19.20 | 20.57 | 20.00 | 23.43 | 17.31 | 21.20 | 24.06 | 22.76 | 21.14 | 23.70 |
| GA-5 | 23.74 | 22.35 | 20.64 | 18.90 | 20.39 | 19.77 | 23.45 | 17.45 | 21.34 | 24.29 | 24.26 | 21.15 | 22.59 |
| GA-6 | 23.44 | 22.29 | 19.76 | 18.92 | 20.35 | 19.44 | 23.28 | 17.53 | 21.55 | 24.20 | 22.54 | 21.10 | 23.53 |
| GA-7 | 23.49 | 22.02 | 20.20 | 18.53 | 19.77 | 19.24 | 23.24 | 17.10 | 20.57 | 23.49 | 22.50 | 20.97 | 23.63 |
| GA-8 | 23.55 | 22.01 | 20.10 | 18.39 | 20.00 | 19.36 | 23.00 | 17.11 | 20.70 | 24.06 | 22.64 | 20.97 | 23.49 |
| GA-9 | 23.64 | 22.10 | 19.76 | 18.46 | 20.28 | 19.00 | 23.06 | 17.26 | 20.73 | 24.85 | 22.96 | 21.12 | 23.52 |
| ABA-1 | 23.88 | 21.92 | 20.64 | 17.53 | 20.72 | 19.03 | 23.16 | 17.28 | 19.00 | 22.09 | 21.06 | 19.79 | 22.03 |
| ABA-2 | 23.35 | 21.12 | 20.49 | 17.37 | 20.37 | 18.57 | 22.69 | 16.82 | 19.15 | 21.16 | 20.68 | 19.35 | 21.43 |
| ABA-3 | 23.47 | 20.91 | 20.44 | 17.32 | 20.21 | 18.32 | 22.46 | 16.62 | 19.45 | 20.86 | 20.27 | 19.17 | 21.25 |
| ABA-4 | 23.16 | 20.63 | 20.19 | 17.15 | 20.10 | 20.19 | 22.17 | 16.44 | 18.62 | 22.20 | 20.20 | 19.03 | 20.95 |
| ABA-5 | 23.03 | 20.82 | 20.28 | 17.00 | 19.81 | 20.16 | 22.12 | 16.54 | 18.85 | 22.03 | 19.97 | 19.05 | 20.93 |
| ABA-6 | 24.43 | 20.77 | 19.53 | 17.01 | 19.75 | 19.92 | 22.04 | 16.22 | 18.91 | 22.07 | 20.12 | 18.91 | 20.77 |
| ABA-7 | 23.48 | 21.17 | 20.48 | 17.31 | 20.24 | 18.63 | 22.39 | 15.76 | 18.38 | 21.09 | 19.82 | 19.09 | 21.30 |
| ABA-8 | 23.43 | 21.01 | 20.42 | 17.31 | 20.23 | 18.59 | 22.51 | 16.60 | 18.55 | 21.18 | 20.93 | 19.56 | 21.67 |
| ABA-9 | 23.60 | 21.25 | 19.97 | 17.30 | 20.85 | 18.96 | 22.79 | 16.76 | 18.62 | 21.39 | 21.28 | 20.04 | 22.07 |
| SA-1 | 22.38 | 21.61 | 19.85 | 18.09 | 20.41 | 19.43 | 23.00 | 17.21 | 19.85 | 23.32 | 22.77 | 21.16 | 22.17 |
| SA-2 | 22.94 | 21.61 | 19.92 | 17.80 | 19.86 | 19.20 | 22.54 | 17.03 | 19.55 | 22.58 | 22.21 | 20.38 | 22.10 |
| SA-3 | 22.42 | 21.31 | 19.61 | 18.03 | 20.03 | 18.76 | 22.35 | 16.87 | 20.04 | 22.52 | 22.12 | 20.20 | 21.86 |
| SA-4 | 22.13 | 21.27 | 19.60 | 18.17 | 20.07 | 19.05 | 22.20 | 16.44 | 19.22 | 22.22 | 21.57 | 20.30 | 21.48 |
| SA-5 | 22.06 | 21.22 | 19.64 | 17.68 | 19.71 | 18.78 | 22.44 | 16.28 | 19.55 | 22.68 | 23.84 | 20.38 | 21.08 |
| SA-6 | 23.64 | 21.43 | 18.99 | 17.79 | 19.83 | 18.46 | 22.03 | 16.31 | 19.49 | 22.34 | 21.53 | 19.97 | 21.27 |
| SA-7 | 21.66 | 20.46 | 19.26 | 17.52 | 19.38 | 18.33 | 21.73 | 16.17 | 18.99 | 22.24 | 21.74 | 20.06 | 21.19 |
| SA-8 | 21.82 | 20.57 | 19.23 | 17.46 | 19.34 | 18.33 | 21.71 | 16.21 | 18.64 | 22.41 | 21.91 | 20.22 | 21.57 |
| SA-9 | 21.86 | 21.01 | 18.22 | 17.44 | 19.96 | 17.33 | 21.95 | 16.27 | 18.88 | 22.88 | 21.97 | 20.30 | 21.67 |
