# Supplementary material for: Selection of Suitable Reference Genes for RT-qPCR Normalization under Abiotic Stresses and Hormone Stimulation in Persimmon (Diospyros kaki Thunb)
Source: PLoS One. 2016 Aug 11;11(8):e0160885. doi: 10.1371/journal.pone.0160885 (PMC4981405; doi:10.1371/journal.pone.0160885)
Supplement: S3 Table — (DOCX) [file pone.0160885.s018.docx]

**Table S3.** Gene expression stability under multiple stresses ranked by geNorm and NormFinder .

| **Group** | **Rank** | **geNorm** | | **NormFinder** | |
| --- | --- | --- | --- | --- | --- |
|  |  | **Gene** | **Stability** | **Gene** | **Stability** |
| Abiotic stress | 1 | *UBC* | 0.30 | *TUA* | 0.181 |
|  | 2 | *RPII* | 0.30 | *UBC* | 0.202 |
|  | 3 | *TUA* | 0.32 | *RPII* | 0.242 |
|  | 4 | *GAPDH* | 0.38 | *PP2A* | 0.243 |
|  | 5 | *PP2A* | 0.47 | *β-TUB* | 0.268 |
|  | 6 | *α-TUB* | 0.52 | *SAND* | 0.286 |
|  | 7 | *SAND* | 0.55 | *GAPDH* | 0.343 |
|  | 8 | *β-TUB* | 0.59 | *α-TUB* | 0.361 |
|  | 9 | *CYP* | 0.65 | *ACT* | 0.481 |
|  | 10 | *RPL13* | 0.70 | *CYP* | 0.559 |
|  | 11 | *ACT* | 0.77 | *EF1-α* | 0.616 |
|  | 12 | *EF1-α* | 0.82 | *RPL13* | 0.724 |
|  | 13 | *F-box* | 0.96 | *F-box* | 0.999 |
| Hormone stimuli | 1 | *α-TUB* | 0.26 | *α-TUB* | 0.089 |
|  | 2 | *PP2A* | 0.26 | *UBC* | 0.185 |
|  | 3 | *GAPDH* | 0.30 | *PP2A* | 0.210 |
|  | 4 | *UBC* | 0.39 | *GAPDH* | 0.271 |
|  | 5 | *TUA* | 0.46 | *SAND* | 0.307 |
|  | 6 | *SAND* | 0.49 | *CYP* | 0.325 |
|  | 7 | *CYP* | 0.53 | *TUA* | 0.330 |
|  | 8 | *EF1-α* | 0.58 | *β-TUB* | 0.385 |
|  | 9 | *F-box* | 0.62 | *F-box* | 0.418 |
|  | 10 | *β-TUB* | 0.67 | *RPL13* | 0.424 |
|  | 11 | *ACT* | 0.70 | *EF1-α* | 0.448 |
|  | 12 | *RPL13* | 0.74 | *ACT* | 0.508 |
|  | 13 | *RPII* | 0.78 | *RPII* | 0.560 |
| Total | 1 | *UBC* | 0.43 | *UBC* | 0.215 |
|  | 2 | *GAPDH* | 0.43 | *PP2A* | 0.273 |
|  | 3 | *CYP* | 0.62 | *SAND* | 0.288 |
|  | 4 | *TUA* | 0.69 | *GAPDH* | 0.316 |
|  | 5 | *β-TUB* | 0.73 | *TUA* | 0.327 |
|  | 6 | *SAND* | 0.77 | *β-TUB* | 0.341 |
|  | 7 | *PP2A* | 0.79 | *CYP* | 0.427 |
|  | 8 | *α-TUB* | 0.82 | *α-TUB* | 0.435 |
|  | 9 | *RPII* | 0.86 | *ACT* | 0.484 |
|  | 10 | *EF1-α* | 0.89 | *EF1-α* | 0.496 |
|  | 11 | *RPL13* | 0.92 | *RPII* | 0.504 |
|  | 12 | *ACT* | 0.96 | *RPL13* | 0.520 |
|  | 13 | *F-box* | 1.05 | *F-box* | 0.667 |
