# Supplementary material for: Selection of Suitable Reference Genes for RT-qPCR Normalization under Abiotic Stresses and Hormone Stimulation in Persimmon (Diospyros kaki Thunb)
Source: PLoS One. 2016 Aug 11;11(8):e0160885. doi: 10.1371/journal.pone.0160885 (PMC4981405; doi:10.1371/journal.pone.0160885)
Supplement: S4 Table — (DOCX) [file pone.0160885.s019.docx]

**Table S4.** Gene expression stability under individual stress ranked by geNorm and NormFinder.

| **Treatments** | **Rank** | **geNorm** | | **NormFinder** | |
| --- | --- | --- | --- | --- | --- |
|  |  | **Gene** | **Stability** | **Gene** | **Stability** |
| Heat | 1 | *CYP* | 0.18 | *UBC* | 0.090 |
|  | 2 | *GAPDH* | 0.18 | *TUA* | 0.126 |
|  | 3 | *TUA* | 0.21 | *GAPDH* | 0.131 |
|  | 4 | *UBC* | 0.24 | *CYP* | 0.169 |
|  | 5 | *RPII* | 0.26 | *F-box* | 0.204 |
|  | 6 | *SAND* | 0.28 | *RPL13* | 0.212 |
|  | 7 | *RPL13* | 0.29 | *PP2A* | 0.230 |
|  | 8 | *PP2A* | 0.31 | *RPII* | 0.233 |
|  | 9 | *β-TUB* | 0.32 | *SAND* | 0.243 |
|  | 10 | *F-box* | 0.35 | *ACT* | 0.283 |
|  | 11 | *ACT* | 0.38 | *β-TUB* | 0.321 |
|  | 12 | *α-TUB* | 0.45 | *EF1-α* | 0.489 |
|  | 13 | *EF1-α* | 0.49 | *α-TUB* | 0.510 |
| Cold | 1 | *RPII* | 0.21 | *UBC* | 0.179 |
|  | 2 | *TUA* | 0.21 | *CYP* | 0.189 |
|  | 3 | *CYP* | 0.25 | *GAPDH* | 0.191 |
|  | 4 | *UBC* | 0.27 | *TUA* | 0.209 |
|  | 5 | *GAPDH* | 0.30 | *β-TUB* | 0.228 |
|  | 6 | *SAND* | 0.37 | *RPII* | 0.230 |
|  | 7 | *β-TUB* | 0.41 | *PP2A* | 0.283 |
|  | 8 | *PP2A* | 0.43 | *SAND* | 0.294 |
|  | 9 | *ACT* | 0.48 | *ACT* | 0.419 |
|  | 10 | *RPL13* | 0.52 | *α-TUB* | 0.435 |
|  | 11 | *α-TUB* | 0.57 | *RPL13* | 0.611 |
|  | 12 | *F-box* | 0.65 | *F-box* | 0.667 |
|  | 13 | *EF1-α* | 0.72 | *EF1-α* | 0.722 |
| NaCl | 1 | *TUA* | 0.19 | *PP2A* | 0.118 |
|  | 2 | *UBC* | 0.19 | *SAND* | 0.168 |
|  | 3 | *RPII* | 0.25 | *UBC* | 0.183 |
|  | 4 | *PP2A* | 0.32 | *TUA* | 0.203 |
|  | 5 | *SAND* | 0.39 | *β-TUB* | 0.282 |
|  | 6 | *GAPDH* | 0.45 | *ACT* | 0.304 |
|  | 7 | *α-TUB* | 0.53 | *RPII* | 0.325 |
|  | 8 | *β-TUB* | 0.58 | *α-TUB* | 0.443 |
|  | 9 | *ACT* | 0.63 | *GAPDH* | 0.509 |
|  | 10 | *CYP* | 0.69 | *CYP* | 0.772 |
|  | 11 | *RPL13* | 0.76 | *EF1-α* | 0.870 |
|  | 12 | *EF1-α* | 0.87 | *RPL13* | 0.967 |
|  | 13 | *F-box* | 0.99 | *F-box* | 1.234 |

| **Treatments** | **Rank** | **geNorm** | | **NormFinder** | |
| --- | --- | --- | --- | --- | --- |
|  |  | **Gene** | **Stability** | **Gene** | **Stability** |
| GA | 1 | *RPL13* | 0.20 | *α-TUB* | 0.103 |
|  | 2 | *PP2A* | 0.20 | *UBC* | 0.118 |
|  | 3 | *GAPDH* | 0.25 | *PP2A* | 0.261 |
|  | 4 | *CYP* | 0.26 | *β-TUB* | 0.263 |
|  | 5 | *ACT* | 0.28 | *TUA* | 0.278 |
|  | 6 | *β-TUB* | 0.29 | *RPL13* | 0.350 |
|  | 7 | *α-TUB* | 0.33 | *SAND* | 0.369 |
|  | 8 | *UBC* | 0.38 | *CYP* | 0.398 |
|  | 9 | *TUA* | 0.44 | *GAPDH* | 0.410 |
|  | 10 | *SAND* | 0.49 | *F-box* | 0.419 |
|  | 11 | *F-box* | 0.55 | *ACT* | 0.445 |
|  | 12 | *RPII* | 0.59 | *RPII* | 0.477 |
|  | 13 | *EF1-α* | 0.65 | *EF1-α* | 0.662 |
| ABA | 1 | *TUA* | 0.19 | *α-TUB* | 0.105 |
|  | 2 | *SAND* | 0.19 | *ACT* | 0.116 |
|  | 3 | *CYP* | 0.21 | *TUA* | 0.120 |
|  | 4 | *PP2A* | 0.23 | *PP2A* | 0.132 |
|  | 5 | *α-TUB* | 0.24 | *CYP* | 0.141 |
|  | 6 | *RPII* | 0.27 | *SAND* | 0.144 |
|  | 7 | *UBC* | 0.29 | *RPII* | 0.201 |
|  | 8 | *GAPDH* | 0.32 | *UBC* | 0.201 |
|  | 9 | *ACT* | 0.36 | *F-box* | 0.204 |
|  | 10 | *β-TUB* | 0.40 | *RPL13* | 0.224 |
|  | 11 | *EF1-α* | 0.43 | *GAPDH* | 0.275 |
|  | 12 | *F-box* | 0.47 | *β-TUB* | 0.279 |
|  | 13 | *RPL13* | 0.52 | *EF1-α* | 0.309 |
| SA | 1 | *UBC* | 0.23 | *UBC* | 0.085 |
|  | 2 | *CYP* | 0.23 | *α-TUB* | 0.094 |
|  | 3 | *α-TUB* | 0.27 | *SAND* | 0.125 |
|  | 4 | *PP2A* | 0.29 | *CYP* | 0.195 |
|  | 5 | *GAPDH* | 0.31 | *β-TUB* | 0.220 |
|  | 6 | *SAND* | 0.33 | *PP2A* | 0.257 |
|  | 7 | *β-TUB* | 0.36 | *F-box* | 0.291 |
|  | 8 | *RPL13* | 0.38 | *RPL13* | 0.330 |
|  | 9 | *F-box* | 0.42 | *GAPDH* | 0.346 |
|  | 10 | *TUA* | 0.46 | *TUA* | 0.417 |
|  | 11 | *EF1-α* | 0.51 | *EF1-α* | 0.450 |
|  | 12 | *ACT* | 0.57 | *ACT* | 0.587 |
|  | 13 | *RPII* | 0.63 | *RPII* | 0.620 |
